# ERdj5 protects goblet cells from endoplasmic reticulum stress-mediated apoptosis under inflammatory conditions

**DOI:** 10.1038/s12276-023-00945-x

**Published:** 2023-02-09

**Authors:** Hyunjin Jeong, Eun-Hye Hong, Jae-Hee Ahn, Jaewon Cho, Jae-Hyeon Jeong, Chae-Won Kim, Byung-Il Yoon, Ja Hyun Koo, Yun-Yong Park, Yoon Mee Yang, Takao Iwawaki, Bruce A. Vallance, Sun-Young Chang, Hyun-Jeong Ko

**Affiliations:** 1 Department of Pharmacy, Kangwon National University, Chuncheon, 24341 Republic of Korea; 2 KNU Researcher training program for developing Anti-Viral Innovative Drugs (BK21 plus), Kangwon National University, Chuncheon, 24341 Republic of Korea; 3 College of Veterinary Medicine, Kangwon National University, Chuncheon, 24341 Republic of Korea; 4 College of Pharmacy, Seoul National University, Seoul, 08826 Republic of Korea; 5 Department of Life Science, Chung-Ang University, Seoul, 06974 Republic of Korea; 6 Division of Cell Medicine, Department of Life Science, Medical Research Institute, Kanazawa Medical University, Uchinada, 920-0293 Japan; 7 BC Children’s Hospital Research Institute, University of British Columbia, Vancouver, BC Canada; 8 College of Pharmacy, Ajou University, Suwon, 16499 Republic of Korea; 9 Global/Gangwon Innovative Biologics-Regional Leading Research Center (GIB-RLRC), Kangwon National University, Chuncheon, 24341 Republic of Korea

**Keywords:** Apoptosis, Acute inflammation, Ulcerative colitis, Endoplasmic reticulum

## Abstract

Endoplasmic reticulum stress is closely associated with the onset and progression of inflammatory bowel disease. ERdj5 is an endoplasmic reticulum-resident protein disulfide reductase that mediates the cleavage and degradation of misfolded proteins. Although ERdj5 expression is significantly higher in the colonic tissues of patients with inflammatory bowel disease than in healthy controls, its role in inflammatory bowel disease has not yet been reported. In the current study, we used *ERdj5*-knockout mice to investigate the potential roles of ERdj5 in inflammatory bowel disease. ERdj5 deficiency causes severe inflammation in mouse colitis models and weakens gut barrier function by increasing NF-κB-mediated inflammation. ERdj5 may not be indispensable for goblet cell function under steady-state conditions, but its deficiency induces goblet cell apoptosis under inflammatory conditions. Treatment of *ERdj5*-knockout mice with the chemical chaperone ursodeoxycholic acid ameliorated severe colitis by reducing endoplasmic reticulum stress. These findings highlight the important role of ERdj5 in preserving goblet cell viability and function by resolving endoplasmic reticulum stress.

## Inflammatory bowel disease: A protective enzyme in intestinal goblet cells

Studies of inflammatory bowel disease (IBD) in mice reveal the role of an enzyme that assists the degradation of mis-folded proteins in the ‘goblet’ cells involved in producing the mucus barrier that lines and protects the interior of the gut. The most common forms of IBD are ulcerative colitis and Crohn’s disease. Researchers in South Korea led by Hyun-Jeong Ko at Kangwon National University, Chuncheon, and Sun-Young Chang at Ajou University, Suwon, compared goblet cell biology in normal mice with mice in which the gene encoding the protein-degrading enzyme ERdj5 had been disabled. This showed that ERdj5 protects goblet cells from a form of stress-mediated cell death that occurs during gut inflammation. The results suggest that drugs modifying the molecular mechanisms underlying ERdj5’s actions could open new avenues towards preventing and treating IBD.

## Introduction

The biological barrier of the gut consists of a monolayer of epithelial cells, immune cells, and the microbiota^1^. The outer surface of the epithelium, which is covered with a sticky layer of mucus, acts as a physical barrier, preventing the entry of noxious substances and enteric pathogens into the body^2^. In the colon, two layers of mucus constitute a thick inner layer that is firmly adherent to the epithelium and impermeable to commensal bacteria and a loose outer layer where the microbiota resides^3^. There are two forms of mucin in the gut: secreted and transmembrane mucins. Secreted mucins, such as mucin 2 (MUC2), form the mucus barrier, whereas transmembrane mucins, such as MUC1, MUC3, MUC4, MUC12, MUC13, and MUC17, are involved in signaling events and barrier defense^4^. Goblet cells produce highly glycosylated mucins to protect the vulnerable intestinal tract^5^. Additionally, colonic mucins provide a niche and energy source for the gut microbiome and act as a physical barrier against pathogens^3,6^. A defective mucus layer with reduced goblet cells is a common characteristic feature of ulcerative colitis (UC)^7^. However, the mechanism underlying goblet cell dysfunction or reduction in patients with UC has not been clearly defined.

The accumulation of misfolded or unfolded proteins within the endoplasmic reticulum (ER) induces the unfolded protein response (UPR) to relieve ER stress^8^. The UPR is a highly conserved mechanism in metazoans and consists of three ER-associated pathways that initiate adaptive transcriptional programs within the nucleus: PKR-like ER-resident kinase (PERK), activated transcription factor 6 (ATF6), and inositol-requiring enzyme 1 (IRE1)/X-box binding protein 1 (XBP-1)^9,10^. Several components of the UPR, including XBP-1, are essential for the biogenesis of the cellular secretory machinery of the exocrine glands^11^. Recently, it was reported that unabated ER stress resulting from the genetic deletion of XBP-1 or misfolded MUC2 within intestinal epithelial cells (IECs), leads to inflammation in the intestinal tract in mice, which is similar to IBD^12,13^. Moreover, it has been shown that polymorphisms in several genes, including XBP-1 and anterior gradient protein-2 (AGR2), increase the risk of both forms of IBD: Crohn’s disease (CD) and UC^13,14^. Elevated ER stress-induced localized intestinal inflammation has been suggested to be associated with the dysfunction of Paneth cells and goblet cells^15^.

Protein disulfide isomerases (PDIs), including endoplasmic reticulum-resident protein 57 (ERp57), endoplasmic reticulum DnaJ domain-containing protein 5 (ERdj5), and AGR2, are a family of ER foldases that catalyze disulfide reduction, oxidation, and isomerization^16^. These factors reduce ER stress by inhibiting the aggregation of unfolded/misfolded proteins in the ER, suggesting a potential role for PDIs as chaperones^17^. PDIs are important for the proper folding and quality control of secreted proteins^18^. MUC2 is a highly glycosylated glycoprotein that acts as a primary defender in IECs and is produced by goblet cells. *MUC2*-knockout (KO) mice develop spontaneous inflammation due to increased exposure to microbial products from the normal flora^19,20^. Considering that MUC2 protein synthesis is regulated by PDIs, defects in AGR2 or ERdj5 may result in misfolded mucin production. Thus, AGR2 deficiency results in the loss of intestinal gel-forming mucus and induces spontaneous inflammation of the intestines in humans and mice^21^.

ERdj5 has disulfide reductase activity that is required for disulfide reduction of proteins to be translocated into the cytoplasm for ER-associated degradation^22^. ERdj5 is also required for the proper folding of several proteins, including the low-density lipoprotein receptor, as well as for the degradation of misfolded proteins^23^. Functional defects in ERdj5 are associated with well-known ER stress-related diseases, such as Parkinson’s and Alzheimer’s disease^24^. Additionally, ERdj5 expression is increased in the salivary glands of patients with Sjögren’s syndrome^25^. Likewise, *ERdj5-*KO mice exhibit increased levels of ER stress in the salivary gland, exhibiting a Sjögren’s syndrome-like phenotype with spontaneous periductal inflammation in the salivary glands^25^. Thus, the ablation of ERdj5 in mice is likely to induce ER stress in highly secretory cells. However, little is known regarding the role of ERdj5 in mucin production and its involvement in IBD development.

Here, we used *ERdj5-*KO mice to investigate the role of ERdj5 in MUC2 production in colonic goblet cells. In the steady state, *ERdj5-*KO mice did not show abnormalities in MUC2 production. However, in the context of dextran sulfate sodium (DSS)-induced colitis, goblet cells in *ERdj5-*KO mice underwent apoptosis due to increased ER stress, resulting in reduced MUC2 production. Our findings revealed that ERdj5 deficiency further exacerbated inflammation in DSS-induced colitis by enhancing barrier breakdown and increasing NF-κB-mediated inflammation. These results suggest that ERdj5 is crucial for maintaining proper goblet cell function.

## Materials and methods

### Animals and treatments

Female C57BL/6 mice were purchased from Koatech (Pyeongtaek, South Korea). *ERdj5-*KO mice (RBRC05508) were purchased from RIKEN BioResource Center (Ibaraki, Japan)^26^. All experiments were approved by the Institutional Animal Care and Use Committee of Kangwon University (IACUC, admission number KW-190319-1). The mice were bred at the Animal Laboratory Center of Kangwon National University. For acute colitis induction, the mice were administered 2% DSS (molecular weight 36–50 kDa; MP Biomedicals, Solon, OH, USA) in drinking water for 5 days, followed by three consecutive days of tap water. Changes in body weight were monitored daily to assess the degree of colitis. On Day 8, colon length was measured, and histological analysis was performed. The mice were administered 500 mg/kg ursodeoxycholic acid (UDCA; Sigma‒Aldrich, St. Louis, MO, USA) once per day via oral gavage beginning on the first day of DSS treatment for a total of 8 days. Additionally, 1 mg/kg SB225002 (Cayman, Ann Arbor, MI, USA), a CXCR2 antagonist, was intraperitoneally (i.p.) injected once daily into DSS-treated mice. Recombinant mouse IL-22 (50 µg/kg, BioLegend, San Diego, CA, USA) was injected i.p. into DSS-treated mice every other day.

### Quantitative real-time PCR and RNA sequencing

Total RNA was extracted from colon tissues using TRIzol reagent (Ambion, Austin, TX, USA) and a tissue-homogenizing matrix kit (Biostar Korea, Daejeon, Korea). The yield and purity of RNA were confirmed using a UV/Vis Nano spectrophotometer (MicroDigital, Seongnam, Korea), and the RNA was reverse-transcribed into cDNA according to the GoScript Reverse Transcription System Technical Manual (Promega, Madison, WI, USA). Quantitative real-time PCR (qRT‒PCR) was performed on a CFX96 Touch Real-Time PCR Detection System (Bio-Rad, Hercules, CA, USA) using a GoTaq qPCR system. All reactions were performed under the following conditions: 39 cycles of predenaturation at 95 °C for 3 min, denaturation at 95 °C for 10 s, and annealing at 60 °C for 30 s and 72 °C for 30 s. The primers used for qPCR were as follows: *m-GAPDH* forward (5′-TGT GTC CGT GGA TCT GA-3′), *m-GAPDH* reverse (5′-CCT GCT TCA CCA CCT TCT TGA T-3′), *m-MUC2* forward (5′-ATG CCC ACC TCC TCA AAG AC-3′), *m-MUC2* reverse (5′-GTA GTT TCC GTT GGA ACA GTG AA-3′), *m-Zo-1* forward (5′-GAC CAA TAG CTG ATG TTG CCA GAG-3′), *m-Zo-1* reverse (5′-TAT GAA GGC GAA TGA TGC CAG A-3′), *m-Cldn1* (Claudin-1) forward (5′-CTG GAA GAT GAG GTG CAG AAG A-3′), *m-Cldn1* reverse (5′-CCA CTA ATG TCG CCA GAC CTG AA-3′), *m-GRP78* (*BiP*) forward (5′-TGT GGT ACC CAC CAA GAA GTC-3′), *m-GRP78* (*BiP*) reverse (5′-TTC AGC TGT CAC TCG GAG AAT-3′), *m-XBP-1s* forward (5′-GAG TCC GCA GGT G-3′), *m-XBP-1s* reverse (5′-GTG TCA GAG TCC ATG GGA-3′), *m-ATF4* forward (5′-CCT GAA CAG CGA AGT GTT GG-3′), and *m*-*ATF4* reverse (5′-TGG AGA ACC CAT GAG GTT TCA A-3′). All primers were synthesized by Macrogen (Seoul, Korea). RNA quality checks, bioinformatics data analysis, and RNA sequencing were performed by Macrogen. The candidate genes were screened based on *P* values < 0.01 and fold-change > 3. The genes of interest were categorized into multiple biological pathways using KEGG^27^. GO clustering analysis was performed using DAVID 6.7 software^28^. The heatmap was visualized using the R package. Microarray data (GSE16879, GSE36807, and GSE47908) were downloaded from the NCBI gene expression omnibus (GEO). The RNA sequencing data are available to access GEO Series number GSE224087. To compare ERdj5 expression in tissue-derived cells, we reanalyzed a previously published dataset^29^ at the EMBL European Bioinformatics Institute (https://www.ebi.ac.uk) under accession number ENSG00000077232.

### ELISA

Interleukin (IL)-6, IL-1β, IL-10, IL-22, CXCL1 and IFN- γ were measured using mouse uncoated ELISA kits (Invitrogen, Vienna, Austria) and mouse IL-22 and CXCL1 Duosets (R&D systems, Minneapolis, MN, USA). Briefly, murine colon tissues were homogenized using a Minilys personal homogenizer (Bertin Technologies, Montigny-le-Bretonneux, France). Tissue homogenates were centrifuged, and the supernatants were analyzed according to the manufacturer’s instructions. The absorbance was measured at 450 nm using a SpectraMax i3 (Molecular Devices, San Jose, CA, USA).

### Cell isolation and flow cytometry

To analyze immune cells in the lamina propria (LP) of the colon, the mouse colon was inverted in a polyethylene tube^30^. To remove mucus and the epithelium, the colon was treated with 1 mM 1,4-dithiothreitol (DTT; Biosesang, Seongnam, Korea) in phosphate-buffered saline (PBS; Corning Inc., Corning, NY, USA) for 10 min at 20 °C and then with 30 mM ethylene-diamine-tetraacetic acid (EDTA; Invitrogen) for 8 min at 20 °C. To isolate immune cells from the LP, colon tissues were incubated with 108 U/ml collagenase IV (Sigma‒Aldrich) at 37 °C for 90 min, and the cells were harvested by shaking for 12 min at 20 °C. After gradient centrifugation with 44–66% Percoll (GE Healthcare Life Sciences, Uppsala, Sweden), mononuclear immune cells were enriched for further analysis. The cells were stained with a combination of the following antibodies: FITC-conjugated anti-CD45 (30-F11; BioLegend), BV421-conjugated anti-CD11b (M1/70; BioLegend), APC-conjugated anti-Ly6C (HK1.4; BD Biosciences), PE-Cy7-conjugated anti-Ly6G (1A8; BD Biosciences), and 7-AAD (BioLegend). For flow cytometry, cells were acquired using a FACS Verse (BD Biosciences, Bergen County, NJ, USA), and the data were analyzed using FlowJo software V10 (BD Biosciences) and 7-AAD-negative and CD45-positive cells. Intestinal epithelial cells (IECs) and nonepithelial cells (non-IECs) were separately obtained on Day 2 following DSS treatment as described previously^31^ and were analyzed to assess ERdj5 expression levels.

### Histology

Colon tissues were rolled and fixed in 10% neutral formalin, and 3 μm paraffin sections were stained with hematoxylin and eosin (H&E). The degree of inflammation was measured by a pathologist using i-Solution Lite (IMT i-Solution Inc., Daejeon, Korea). The pathological grade is expressed as a percentage^32^: 0, normal; g1, intestinal gland loss ≤ 25% of the LP mucosae and slightly increased inflammatory cell infiltration of the LP; g2, intestinal gland loss ≥ 25% or ≤ 50% and significantly increased inflammatory cell infiltration; and g3, intestinal gland loss ≥ 50% with severe erosion of inflammatory cells. To visualize mucin and goblet cells, colon sections were stained with a Periodic acid Schiff (PAS) stain kit (Abcam, Cambridge, UK) according to the manufacturer’s instructions. The number of goblet cells per crypt was determined using a Nikon Eclipse Ts2 (Nikon, Minato, Tokyo, Japan).

### Immunofluorescence staining

Colon tissue Section (5 µm thick) were deparaffinized, and antigens were retrieved using a microwave and 10 mM sodium citrate buffer (Donginbiotech Co., Seoul, Korea). After being blocked with 1% bovine serum albumin (BSA; MP Biomedicals) in PBS, the tissues were stained with primary antibodies overnight at 4 °C. After being washed with PBS, the tissues were incubated with secondary antibodies for 2 h at 4 °C. The slides were mounted with a mounting solution containing 4′,6-diamidino-2-phenylindole (DAPI; Vector, Burlingame, CA, USA) for nuclear staining.

For organoid staining, the colonoids were fixed with 4% paraformaldehyde (Invitrogen) for 1 h at 20 °C and permeabilized with 2% Triton X-100 (Sigma‒Aldrich) in PBS for 2 h at 37 °C. After being blocked with 3% BSA in PBS containing 1% Triton X-100 for 2 h at 20 °C, the colonoids were incubated with primary antibodies overnight at 4 °C. The colonoids were incubated with secondary antibodies for 2 h at 37 °C and mounted with antifade mounting medium containing DAPI. To evaluate cell apoptosis, a TUNEL assay was performed using an ApopTag Fluorescein In Situ Apoptosis Detection Kit (Merck, Kenilworth, NJ, USA) according to the manufacturer’s instructions. Images were obtained using a confocal laser scanning microscope (LSM880; Carl Zeiss, Göttingen, Germany) at the Central Laboratory of Kangwon National University and analyzed with Zen software (Carl Zeiss).

The samples were stained with primary antibodies, including rabbit anti-mouse MUC2 (PA5-79702, 1:200; Invitrogen), rabbit anti-mouse CLCA1 (clone EPR12254-88, 1:200; Abcam), Alexa Fluor 647-conjugated rat anti-mouse E-cadherin (clone DECMA-1, 1:200; BioLegend), rabbit anti-mouse ZO-1 (61-7300, 1:250; Invitrogen), mouse anti-mouse Claudin-1 (clone 2H10D10, 1:250; Invitrogen), rabbit anti-mouse NF-κB (clone D14E12, 1:500; Cell Signaling Technology), rabbit anti-mouse myeloperoxidase (MPO; PA5-16672, 1:200; Invitrogen), APC-conjugated Ly6G (clone 1A8, 1:200; BD Biosciences), and goat anti-mouse lysozyme C (sc-27958, 1:200; Santa Cruz Biotechnology, Dallas, TX, USA). The secondary antibodies used were anti-mouse IgG H&L Alexa Fluor 488 (ab150105, 1:500; Abcam), anti-rabbit IgG (H + L) Alexa Fluor 594 (A-11012, 1:400; Invitrogen), anti-rabbit IgG H&L Alexa Fluor 647 (ab150079, 1:400; Abcam), and anti-goat IgG (H + L) Alexa Fluor 555 (A32816, 1:400; Invitrogen).

### Colon organoid culture

Colon organoids were cultured as previously described^33^. Briefly, the colon was opened longitudinally and treated with penicillin‒streptomycin (Gibco, Carlsbad, CA, USA) and gentamycin (Gibco) at 4 °C for 30 min to remove microbiota, and a cell recovery solution (Corning) was added and incubated at 4 °C for 30 min. Using curved forceps, villi were scratched and centrifuged at 800 rpm and 4 °C. After being washed with advanced DMEM/F12 (Gibco), the harvested crypts were counted. After being mixed 1:1 with Matrigel (Corning) and advanced DMEM/F12 containing penicillin‒streptomycin, Glutamax (Gibco), and HEPES (Gibco), the media containing the crypts was plated in 24-well plates and filled with 50% L-WRN growth media containing DMEM/F12, 20% fetal bovine serum (FBS, Gibco), penicillin‒streptomycin, B27 (Invitrogen), N2 (Invitrogen), 10 mM nicotinamide (Sigma), 1.25 mM N-acetylcysteine (Sigma), 16.7 pM mEGF (Invitrogen), 10 μM P38i (Sigma), 0.5 μM A83-01 (Tocris, Bristol, UK), and 10 μM Y-27632 (Abmole, Houston, TX, USA). In 24-well plates, the crypt seeding density was 100 crypts/well. Colonoids were passaged to a maximum of passage 3 and used for experiments when they stopped growing and the inner necrotic core started to darken^34^, which usually occurred on Day 5 after Passage 3. We validated the results using three replicate experiments and proceeded with 200 colonoids per experiment. To mimic inflammatory conditions, colonoids were treated with Pam_3_CSK_4_ (InvivoGen, San Diego, CA, USA), an agonist of Toll-like receptor (TLR)2/TLR1, for 48 h at 37 °C on Day 5.

### Citrobacter rodentium infection

*C. rodentium* (DBS100) was cultured overnight in a shaking incubator at 200 rpm and 37 °C in Luria-Bertani (LB) broth one day before infection. The mice were infected by oral gavage with 0.1 ml of LB broth containing 2.5 × 10^8^ CFU of streptomycin-resistant *C. rodentium*^35^. The mice were monitored and weighed daily for symptoms of distress or illness. On Day 14 postinfection, CFUs were measured in the colon and cecum. Tissue homogenates were serially diluted and cultured overnight at 37 °C in LB agar containing streptomycin. On Day 10 postinfection, mouse colon tissue was collected for immunofluorescence staining of goblet cell apoptosis.

### Western blotting

Colon tissues were homogenized in PRO-PREP™ Protein Extraction Solution (Intron, Seongnam, Korea) and mixed with 5× sample buffer (Genscript, Piscataway, NJ, USA) containing a protease inhibitor (Gendepot, Katy, TX, USA) and phosphatase inhibitor (Gendepot). The protein concentration was quantified using the BCA protein assay (Abbkine, Wuhan, China). The antibodies used for western blotting were PERK (clone C33E10), p-PERK (clone 16F8), eIF-2α (9722 S), p-eIF-2α (9721 S), GRP78/BiP (clone C50B12), CHOP (clone L63F7), IKK (2682 S), p-IKK (clone 16A6), IκB (9242 S), NF-κB p65 (clone D14E12), p-NF-κB p65 (clone 93H1), and anti-rabbit IgG-HRP (7074P2), all of which were purchased from Cell Signaling Technology (Danvers, MA, USA); ATF4 (clone H-290), β-actin (clone C4), and ERdj5 (clone 66.7), which were purchased from Santa Cruz Biotechnology; and anti-mouse IgG-HRP (ADI-SAB-100-J), which was purchased from Enzo Life Sciences (NY, USA). Protein bands were visualized using a chemiluminescence detection system for horseradish peroxidase (GBioscience, St. Louis, MO, USA).

The lysates of MODE-K cells were used to determine the expression of ER stress marker proteins, including PERK, p-PERK, eIF-2α, and p-eIF-2α, using western blotting. MODE-K cells were activated with Pam_3_CSK_4_ for a specified time, and NF-κB pathway signaling was analyzed. MODE-K cells were treated with 5 µM GSK 2606414 (Tocris), a PERK inhibitor, for 2 hr before Pam_3_CSK_4_ treatment.

### Construction of ERdj5-deficient MODE-K cells

The mouse intestinal epithelial cell line MODE-K^36^ was cultured in RPMI-1640 (Corning) supplemented with 10% FBS, Glutamax, sodium pyruvate, and MEM NEAA (all from Gibco). LentiCRISPRv2, which was a gift from Feng Zhang, was purchased from Addgene (Addgene plasmid #52961; http://n2t.net/addgene:52961; RRID: Addgene_52961)^37^. The vector was digested using the restriction enzyme BsmBI (Enzynomics, Daejeon, Korea). After the vector was cut, gel extraction (Real Biotech, Taipei, Taiwan) was performed. The cleaved vector and annealed oligo were ligated (Real Biotech, Beijing, China). sgRNA (genome target site: 5′-GTA CAG TGG GGG CTA AAT CA-3′) targeting the mouse ERdj5 gene was cloned downstream of the U6 promoter, which was cut by the BsmB1 enzyme to generate a lentivirus vector. After ligation, the cells were transformed with Stbl3. The next day, a single colony was chosen, placed in LB broth and grown overnight in a shaking incubator. Plasmid DNA was extracted from liquid bacterial cultures using a Miniprep kit (Qiagen, Hilden, Germany). HEK 293FT cells were transfected with Lipofectamine 2000 (Life Technologies, Carlsbad, CA, USA) using purified plasmid DNA and packaging vectors. psPAX2 (Addgene plasmid #12260; http://n2t.net/addgene:12260; RRID: Addgene_12260) and pMD2.G (Addgene plasmid #12259; http://n2t.net/addgene:12259; RRID: Addgene_12259) were gifts from Didier Trono. The medium was changed after 6 h, and the virus was harvested after 72 h. MODE-K cells were infected with virus in medium containing 6 mg/ml polybrene (Sigma). After 8 h, the medium was replaced with complete medium, and cells were cultured with 6 mg/ml puromycin (Gibco) to select virus-infected cells. The deletion of ERdj5 was confirmed by western blot analysis.

### Statistical analysis

Data analysis was performed using GraphPad Prism 9 (San Diego, California, USA) and FlowJo V10 software. Differences between the two groups were determined using Student’s *t* test. One-way analysis of variance (ANOVA) followed by post hoc tests (Tukey’s multiple comparisons test) and two-way ANOVA followed by post hoc tests (Bonferroni’s multiple comparisons test) were used to compare data from more than two groups. A *P* value < 0.05 was considered significant. The data are representative of three independent experiments.

## Results

### ERdj5 expression is upregulated in UC colons

**Fig. 1:**
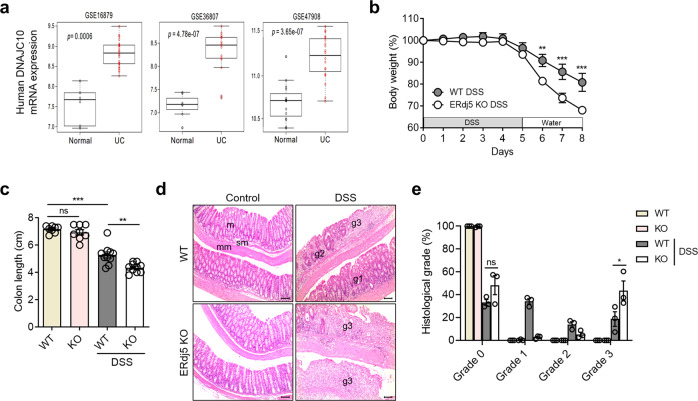
ERdj5 deficiency exacerbates DSS-induced colitis in a murine model. **a** *ERdj5* (*DNAJC10*) mRNA expression levels in normal controls or patients with ulcerative colitis (UC) were analyzed in the public GEO database. **b**–**e** Mice were administered 2% DSS in drinking water for 5 days and subsequently switched to normal water (*n* = 8–10 per group). **b** Body weight. **c** Colon length on Day 8. **d** Representative histopathological features of the colon in H&E-stained sections (×100, scale bar: 100 µm); m, mucosa; sm, submucosa; mm, muscular layer; pathological grade (g1, g2, g3). **e** Measurement of the length with different grading scores of inflammation in the colon. The data are representative of three independent experiments, and the values are expressed as the mean ± SEM; **P < 0.01 and ***P < 0.001; ns, not significant. Two-way ANOVA followed by Bonferroni’s test for (**b**) and one-way ANOVA followed by Tukey’s test for (**c**).

Although ERdj5 is highly expressed in the colon in humans and mice^38^, few reports have suggested a role for ERdj5 in gut physiology and pathogenic colitis. Gene expression profile analysis was performed using BRB-array tools with GEO data (GSE16879, GSE36807, and GSE47908). To examine the expression levels of the indicated genes, a comparative analysis was performed with R software. *ERdj5* mRNA expression levels were significantly higher in the colon tissues of patients with UC than in those of normal controls (Fig. 1a). *ERdj5* mRNA levels were positively correlated with *MUC2* mRNA levels in patients with UC (Supplementary Fig. 1a). In addition, oral DSS treatment dramatically increased the protein expression of ERdj5 in murine colon tissue (Supplementary Fig. 1b). On Day 2 of DSS treatment, ERdj5 expression increased significantly in IECs but not in non-IECs in WT mice (Supplementary Fig. 1c-f). In addition, MUC2 expression was reduced in the colonic IECs of *ERdj5-*KO mice on Day 2 of DSS treatment, and a similar positive correlation between *MUC2* and *ERdj5* expression was observed in the WT CON/WT DSS groups as in the patient data (Supplementary Fig. 1c, g, h). We used single-cell transcriptome analysis to further confirm that *ERdj5* was primarily expressed in the goblet cells of the human large intestine^29^.

### ERdj5 deficiency induces more severe inflammation in DSS-induced colitis

To determine whether ERdj5 plays a protective or detrimental role in colonic inflammation, DSS-induced colitis was evaluated in wild-type (WT) and *ERdj5-*KO mice. Although no apparent colonic inflammation was observed in *ERdj5-*KO mice under steady-state conditions, the administration of 2% DSS for 5 days significantly decreased body weight and colon length compared to those of WT mice (Fig. 1b, c). The level of tissue damage in the entire colon was assessed by H&E staining, and DSS-treated *ERdj5-*KO mice had more severe intestinal gland loss and inflammatory cell infiltration than WT mice (Fig. 1d). Histological analysis was performed, and the degree of inflammation is expressed as a percentage of the length combined with the degree of severity of the entire colon. A total of 18.7% of WT and 43.5% of KO mice had Grade 3 colonic inflammation, with significant loss of intestinal glands and severe deposition of inflammatory cells on the mucous membrane plate (Fig. 1e).

**Fig. 2:**
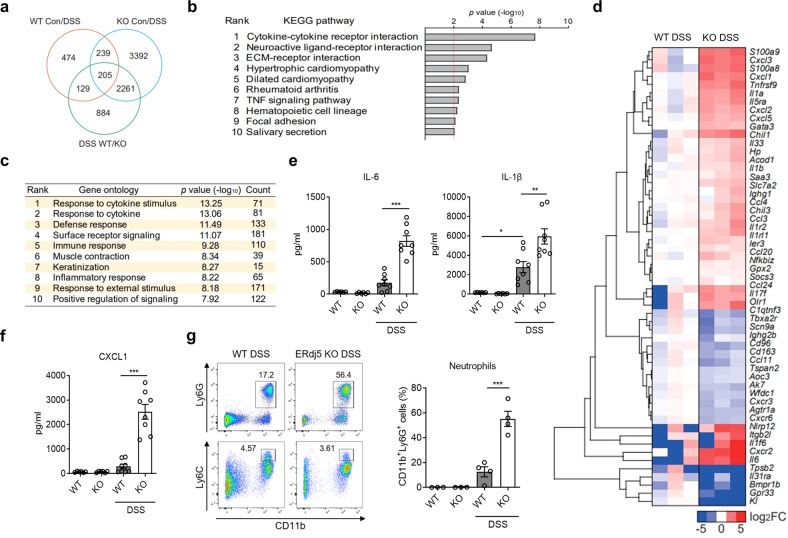
RNA-seq reveals an altered gene expression profile for inflammation in the absence of ERdj5. **a** Venn diagram. The number of differentially expressed genes (DEGs) from colon samples extracted from 8-week-old female WT or *ERdj5-*KO mice (*n* = 3 per group) treated with or without 2% DSS. **b** KEGG pathway analysis of DEGs in colon samples extracted from 8-week-old female WT or *ERdj5-*KO mice (*n* = 3 per group) treated with or without 2% DSS. Pathways with statistical significance (*P* > 0.01) are shown. **c** GO analysis of major signaling pathways identified by RNA sequencing using the DAVID bioinformatics database. The top 10 pathways based on *P* values are shown, along with the number of genes with the respective ontology. **d** Heatmap of the DEGs associated with inflammation. The log_2_ ratios of *ERdj5* KO DSS/WT DSS are presented (blue, underexpression; red, overexpression). Values over 5 or under −5 were rounded. **e** Levels of IL-1β and IL-6 in colon homogenates (*n* = 6–8 per group). **f** Level of CXCL1 in colon homogenates (*n* = 6–8 per group). **g** Representative flow cytometric analysis of CD11b^+^Ly6G^+^ neutrophils and CD11b^+^Ly6C^+^ monocytes among pregated live CD45^+^IA-IE^-^CD11c^-^ cells in the LP of the colon. The percentage of CD11b^+^Ly6G^+^ neutrophils is expressed as the mean ± SEM (*n* = 3–4 per group). *P < 0.05, **P < 0.01, ***P < 0.001; one-way ANOVA followed by Tukey’s test.

To further investigate the role of ERdj5 in colitis, colon samples from DSS-treated mice were obtained, and RNA sequencing was performed. Numerous changes were observed in the differentially expressed genes (DEGs) in DSS-treated *ERdj5-*KO mice compared to DSS-treated WT mice (Fig. 2a, b). An extensive analysis of GO terms using the DAVID bioinformatics database showed that many genes with particular ontologies, including the response to cytokines, surface receptor signaling, and the inflammatory response, were significantly altered in the colons of DSS-treated *ERdj5-*KO mice compared to WT mice (Fig. 2b, c). A heatmap of the DEGs associated with inflammation indicated that several cytokines and chemokines associated with severe inflammation, including *Cxcl1, Cxcl2, Il1b, and Il6*, were more highly expressed in the colon samples of DSS-treated *ERdj5-*KO mice than in those of WT mice (Fig. 2d, Supplementary Fig. 2a). We also confirmed elevated protein expression levels of IL-1β, IL-6, and CXCL1 in the distal colon tissues of DSS-treated *ERdj5-*KO mice (Fig. 2e, f). Moreover, neutrophil-related genes, including *S100a8*, *S100a9*, and *Saa3*^39,40^, were increased in the inflamed colon tissues of DSS-treated *ERdj5-*KO mice compared to those of WT mice (Fig. 2d). Furthermore, a significant correlation between *ERdj5* and *S100A8/A9* was found in UC patients following mRNA analysis of data from a public database (Supplementary Fig. 1a). We found that Ly6G^+^ neutrophil infiltration was elevated in the colons of DSS-treated *ERdj5-*KO mice (Fig. 2g, Supplementary Fig. 3a, b). The transcription levels of *Arg1* and *Nos2*, which are increased in neutrophils^41,42^, were also increased in DSS-treated *ERdj5-*KO mice (Supplementary Fig. 2b). However, the inhibition of neutrophil migration by a CXCR2 antagonist did not alleviate DSS-induced colitis in *ERdj5-*KO mice (Supplementary Fig. 3c). Collectively, these results demonstrate that ERdj5-deficient mice exhibited more severe inflammation with profound neutrophil infiltration than WT mice under DSS-induced colitis conditions; however, the inhibition of neutrophil infiltration alone did not attenuate severe colitis in these mice.

### ERdj5 deficiency enhances goblet cell death in response to stress

**Fig. 3:**
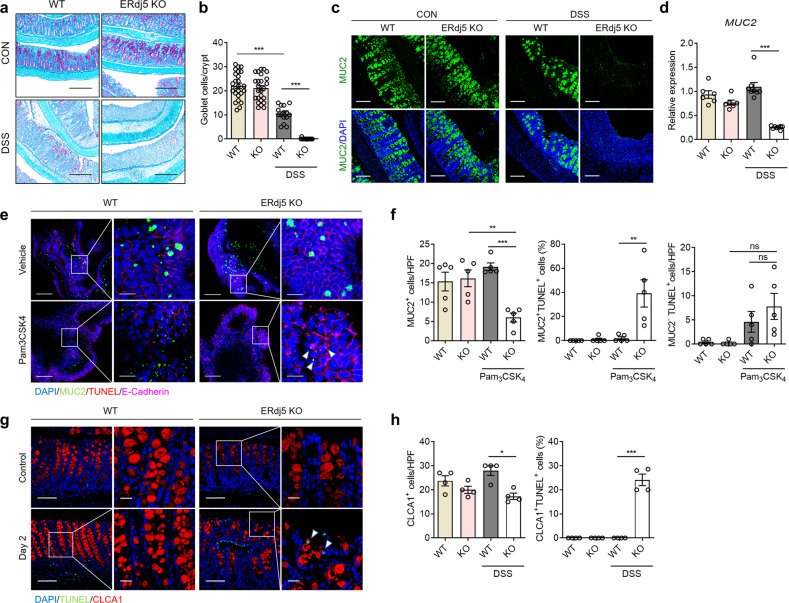
ERdj5 deficiency decreases mucin secretion and goblet cells in response to inflammatory signals. **a** Periodic acid Schiff (PAS) staining of the colons of WT and *ERdj5-*KO mice (×100, scale bar 500 µm; *n* = 6 per group). **b** Goblet cell count per crypt in PAS images (*n* = 15–25 per group). **c** Representative immunofluorescence images of MUC2 (green) in colon tissues, with DAPI (blue) for nuclear staining (×200, scale bar 200 µm). **d** Level of *MUC2* mRNA expression (*n* = 6–8 per group). **e** Representative immunofluorescence images of colonoids originating from WT or *ERdj5-*KO mice treated with vehicle or Pam_3_CSK_4_. MUC2 (green), TUNEL (red), E-cadherin (magenta), and DAPI (blue) (left panel, ×200 and right panel, ×800). **f** MUC2^+^ cell counts per high-power field (HPF) and the percentage of TUNEL-positive cells among MUC2^+^ and MUC2^-^ cells determined in colonoid images (*n* = 5 per group). **g** Representative immunofluorescence images of colon tissues from WT or *ERdj5-*KO mice on Day 2 following DSS treatment. CLCA1 (red), TUNEL (green), and DAPI (blue) (left panel, ×200 and right panel, ×630). **h** CLCA1^+^ cell count per HPF and TUNEL-positive percentages among CLCA1^+^ cells from colon tissue images (*n* = 4 per group). **e**, **g** Scale bars correspond to 100 μm and 20 μm, respectively. The data are representative of three independent experiments, and the values are expressed as the mean ± SEM. *P < 0.05, **P < 0.01, ***P < 0.001; one-way ANOVA followed by Tukey’s test.

In association with severe inflammation, intestinal goblet cell density was markedly reduced in the colons of DSS-treated *ERdj5-*KO mice, as shown by PAS staining of heavily glycosylated proteins (Fig. 3a, b). We further examined MUC2 protein expression in the colon by immunofluorescence staining using MUC2-specific antibodies (Fig. 3c). PAS-positive cells and *MUC2* expression were comparable in the colons of *ERdj5* KO and WT mice without DSS administration; however, these levels were significantly decreased in the colons of DSS-treated *ERdj5-*KO mice compared to DSS-treated WT mice (Fig. 3b–d). These results suggest that severe inflammation caused by ERdj5 deficiency leads to the loss of goblet cells.

To assess whether the increased inflammation in the colons of *ERdj5-*KO mice was directly associated with the increased apoptosis of goblet cells, we evaluated apoptosis in MUC2-producing cells using colon organoids derived from WT and *ERdj5-*KO mice. There was no remarkable structural differences in colonoids obtained from the WT group and *ERdj5* KO groups without stimulation (Supplementary Fig. 4a). However, treating mature colonoids with Pam_3_CSK_4_, a TLR2 ligand, partially ruptured the structure of those in the *ERdj5* KO group, while colonoids in the WT group did not rupture (Supplementary Fig. 4b). In addition, an increase in CXCL1 production was observed in the culture supernatant of *ERdj5* KO organoids compared to WT organoids following Pam_3_CSK_4_ treatment (Supplementary Fig. 4c). Immunofluorescence staining of colonoids confirmed that the number of MUC2-producing goblet cells was lower in *ERdj5-*KO mice than in WT mice following stimulation with Pam_3_CSK_4_ (Fig. 3e, f). Intriguingly, MUC2^+^TUNEL^+^ cells were significantly increased in Pam_3_CSK_4_-treated *ERdj5* KO colonoids compared with Pam_3_CSK_4_-treated WT colonoids, suggesting that TLR2 stimulation induced apoptosis in ERdj5-deficient goblet cells (Fig. 3f). Most distal colon cells obtained from *ERdj5-*KO mice on Day 8 after DSS treatment were severely damaged (Fig. 3a–d); therefore, it was difficult to evaluate sections with similar levels of inflammation. Thus, we compared colon tissues obtained on Day 2 after DSS treatment. An increased proportion of goblet cells expressing CLCA1 and MUC2 underwent apoptosis in the distal colons of DSS-treated *ERdj5-*KO mice, even though there was no evident inflammation (Fig. 3g, h, and Supplementary Fig. 5a, b). Next, we analyzed UPR gene expression in isolated mouse IECs on Day 2 of DSS treatment and observed a significant increase in *GRP78* and *CHOP* expression in *ERdj5-*KO IECs but not in WT IECs (Supplementary Fig. 5c). Thus, we hypothesize that early upregulation of UPR gene expression on Day 2 of DSS treatment drives goblet cell apoptosis. However, lysozyme^+^ Paneth cells, which are highly secretory cells in the the small intestine, were not affected in *ERdj5-*KO mice, even under DSS insult (Supplementary Fig. 5d, e). Collectively, these results suggest that ERdj5 deficiency may trigger apoptosis in secretory epithelial cells, especially goblet cells, and further exacerbate intestinal inflammation.

### ERdj5 helps maintain gut barrier function by enhancing tight junctions

**Fig. 4:**
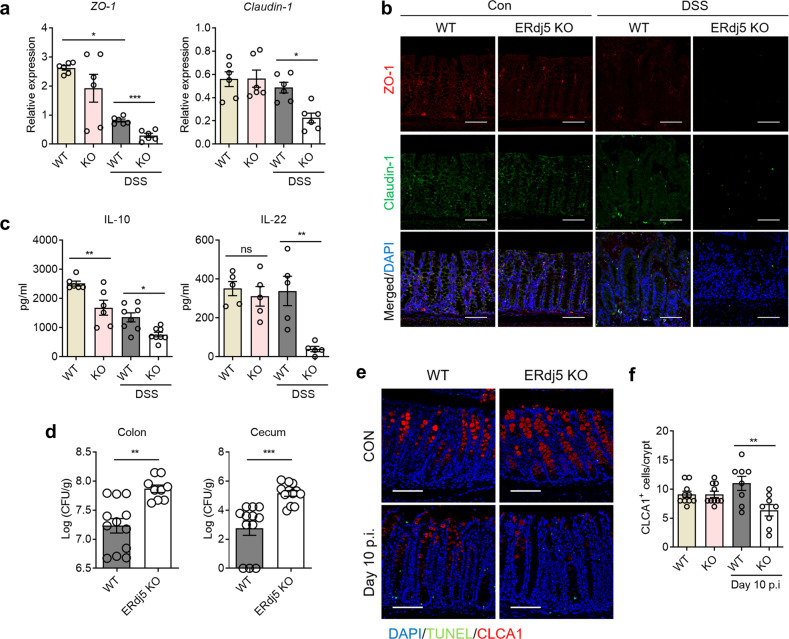
ERdj5 contributes to the maintenance of intestinal barrier integrity. **a** *Zo-1* and *Cldn1* mRNA expression in the colon tissues of WT or *ERdj5-*KO mice treated with DSS (*n* = 6 per group). **b** Immunofluorescence images of colon tissue stained for the tight junction proteins ZO-1 (red) and Claudin-1 (green) (×200, scale bar 100 µm). **c** IL-10 and IL-22 expression in colon tissue homogenates (*n* = 5–8 per group). **d** Mice were infected with 2.5 × 10^8^ CFU of *C. rodentium*. On Day 14 postinfection, CFUs in the colon and cecum were measured (*n* = 9–12 per group). **e** Representative immunofluorescence images of colon tissues from WT or *ERdj5-*KO mice 10 days after *C. rodentium* infection. CLCA1 (red), TUNEL (red), and DAPI (blue) (×200, scale bar 100 µm). **f** CLCA1^+^ cell counts per crypt of WT and *ERdj5-*KO mice in **e** (*n* = 10–12 per group). The data are representative of three independent experiments, and the values are expressed as the mean ± SEM. *P < 0.05, **P < 0.01, ***P < 0.001; one-way ANOVA followed by Tukey’s test for **a**, **c**, **f** and Student’s *t test* for **d**.

Tight junctions act as barriers in the intestinal epithelium and prevent microbial intrusion^43^. In patients with IBD, intestinal barrier destruction is common, which worsens inflammatory pathology^44^. Thus, we postulated that ERdj5 plays a role in the maintenance of gut barrier integrity. The levels of typical tight junction proteins, such as zonula occludens-1 (ZO-1) and Claudin-1, were decreased in the colons of *ERdj5-*KO mice compared to WT mice after DSS administration. However, no changes were detected in Zo-1 or Claudin-1 expression in *ERdj5-*KO colons compared to WT colons under normal conditions (Fig. 4a). The immunofluorescence results for ZO-1 and Claudin-1 were similar to the mRNA results (Fig. 4b). In addition, we found that the expression of IL-10 and IL-22, which are critical for the maintenance of intestinal homeostasis^45^, was significantly lower in *ERdj5-*KO mice than in WT mice after DSS administration (Fig. 4c). Treatment with recombinant IL-22 ameliorated DSS-induced colitis in WT mice^46^ but not in *ERdj5-*KO mice (Supplementary Fig. 3d). Thus, we infer that IL-22 treatment does not rescue goblet cell apoptosis in *ERdj5-*KO mice.

We next assessed whether the reduction in gut barrier function and mucin production due to ERdj5 deficiency impacted protection against enteric bacteria. *ERdj5-*KO mice were orally infected with 2.5 × 10^8^ CFU of *C. rodentium* and monitored for 2 weeks to assess morbidity and mortality. No infection-related deaths were recorded in either group; however, *ERdj5-*KO mice exhibited delayed clearance of *C. rodentium* in the cecum and colon 14 days postinfection (Fig. 4d). The levels of inflammatory cytokines, including IFN-γ, IL-1β, and IL-6, were increased in *ERdj5-*KO mice following *C. rodentium* infection (Supplementary Fig. 6). FITC-dextran levels were significantly increased in DSS-treated *ERdj5-*KO mouse serum compared to DSS-treated WT mice, as assessed by a FITC-dextran permeability assay^47^, suggesting that *ERdj5-*KO mice had impaired gut barrier function (Supplementary Fig. 7). We also analyzed the number of goblet cells, since *C. rodentium* infection reportedly decreases the goblet cell count^48^. Although there was no difference in CLCA1^+^ cells in the colons of uninfected mice in the two groups, the number of CLCA1^+^ goblet cells was significantly reduced in the colons of *ERdj5-*KO mice on Day 10 postinfection (Fig. 4e, f). Taken together, these results suggest that ERdj5 is required for proper mucin secretion from goblet cells and the maintenance of gut barrier integrity under stressful conditions.

### ERdj5 deficiency activates NF-κB-dependent inflammatory signals by enhancing the UPR, which leads to epithelial cell apoptosis

**Fig. 5:**
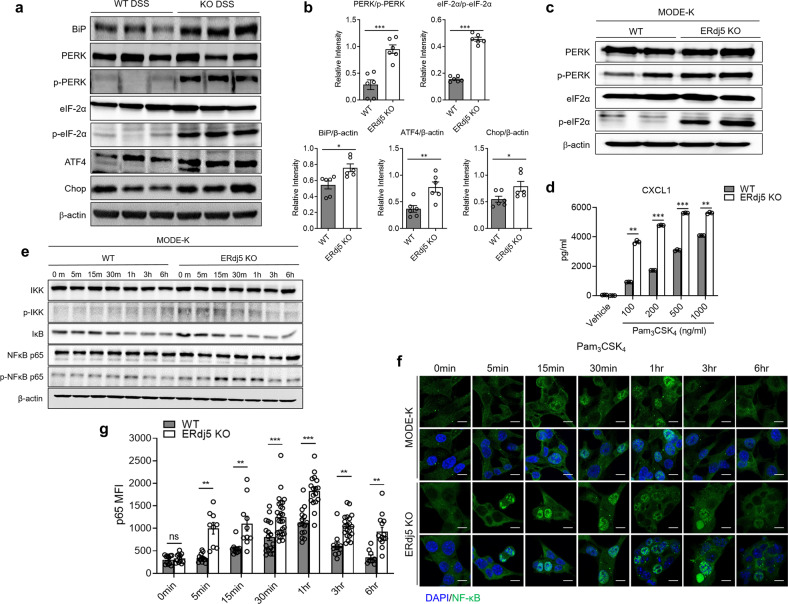
ERdj5 deficiency causes ER stress and inflammation mediated by NF-κB activation. **a** Expression profile of ER stress-related proteins in the colons of WT or *ERdj5-*KO mice treated with DSS (*n* = 6 per group). **b** Relative band intensities are expressed as the mean ± SEM. **c** ER stress proteins in *ERdj5-*KO MODE-K cells altered by the CRISPR/Cas9 system. **d** The level of CXCL1 in *ERdj5-*KO MODE-K cells after Pam_3_CSK_4_ treatment (100, 200, 500, and 1000 ng/ml) for 6 h (*n* = 3 per group). **e** The protein expression of NF-κB pathway factors in MODE-K cells treated with Pam_3_CSK_4_ (1 µg/ml) for 0, 5, 15, 30, 60, 180, and 360 min. **f** Nuclear translocation of NF-κB/p65 in *ERdj5-*KO MODE-K cells at the indicated time points after Pam_3_CSK_4_ treatment (1 µg/ml). **f** Immunofluorescence image (×200, scale bar 10 µm). **g** The mean fluorescence intensity of NF-κB/p65 (green) is shown (*n* = 9~27 per group). The data are representative of three independent experiments, and the values are expressed as the mean ± SEM. *P < 0.05, **P < 0.01, ***P < 0.001; Student’s *t test*.

DSS administration increased the transcription of several genes associated with protein processing in the ER, including *Sec31b, Prkn, Cryab*, and *Tram1l1*, in the colon tissues of *ERdj5-*KO mice (Supplementary Fig. 2b), suggesting that the ablation of ERdj5 altered abnormal protein processing. Considering that it was previously reported that ERdj5 regulates the UPR by inhibiting the phosphorylation of eukaryotic initiation factor 2α (eIF2α)^49^, we next assessed the levels of phosphorylated eIF2α and PERK and found that they were higher in the colon tissues of *ERdj5-*KO mice than in those of WT mice (Fig. 5a, b). In addition, a significant increase was observed in the levels of GRP78 and other PERK pathway-related proteins, including ATF4 and CHOP, in *ERdj5-*KO mice. The mRNA expression levels of *ATF4, XBP1s*, and *GRP78* were also higher in DSS-treated *ERdj5-*KO mice than in DSS-treated WT mice (Supplementary Fig. 8a–c). *XBP1* mRNA levels were positively correlated with *ERdj5* mRNA levels in UC patients (Supplementary Fig. 1a). To further assess changes in the molecular expression of UPR factors induced by ERdj5 ablation, we generated *ERdj5-*KO MODE-K cells using CRISPR/Cas9 (Supplementary Fig. 9a). The loss of ERdj5 led to a marked increase in the levels of phosphorylated eIF2α, indicating an increase in the PERK/eIF2α branch of the UPR (Fig. 5c).

Previous studies have suggested that an elevated UPR can increase NF-κB-mediated signaling via stimulation with TLR ligands^50^. Increased intestinal ER stress may increase inflammation by elevating NF-κB-dependent chemokine expression. To test our hypothesis, we assessed the expression level of CXCL1 in *ERdj5-*KO MODE-K cells following stimulation with Pam_3_CSK_4_. *ERdj5-*KO MODE-K cells secreted higher levels of CXCL1 than WT MODE-K cells in a dose-dependent manner (Fig. 5d). These results suggest that ERdj5 deletion in IECs increases CXCL1 secretion in response to TLR2 stimulation.

To clarify the mechanism of TLR2-induced CXCL1 production in ERdj5-deficient cells, we examined the nuclear translocation of NF-κB. Enhanced phosphorylation of IκB kinase (IKK) and early degradation of inhibitor of NF-κB alpha (IκBα) in *ERdj5-*KO MODE-K cells led to an increase in the level of phosphorylated NF-κB p65 (Fig. 5e). Accordingly, nuclear localization of NF-κB p65 in *ERdj5-*KO MODE-K cells was significantly increased after Pam_3_CSK_4_ treatment at early time points (Fig. 5f, g), suggesting that ERdj5 deficiency enhanced NF-κB activation. In addition, treatment of *ERdj5*-KO MODE-K cells with a PERK inhibitor inhibited NF-κB signaling following TLR2 stimulation (Supplementary Fig. 9b). Collectively, these results suggest that enhanced ER stress in the absence of ERdj5 sensitizes CXCL1 production via TLR2 stimulation via early and enhanced NF-κB activation.

### The chemical chaperone UDCA ameliorates DSS-induced colitis in the absence of ERdj5

**Fig. 6:**
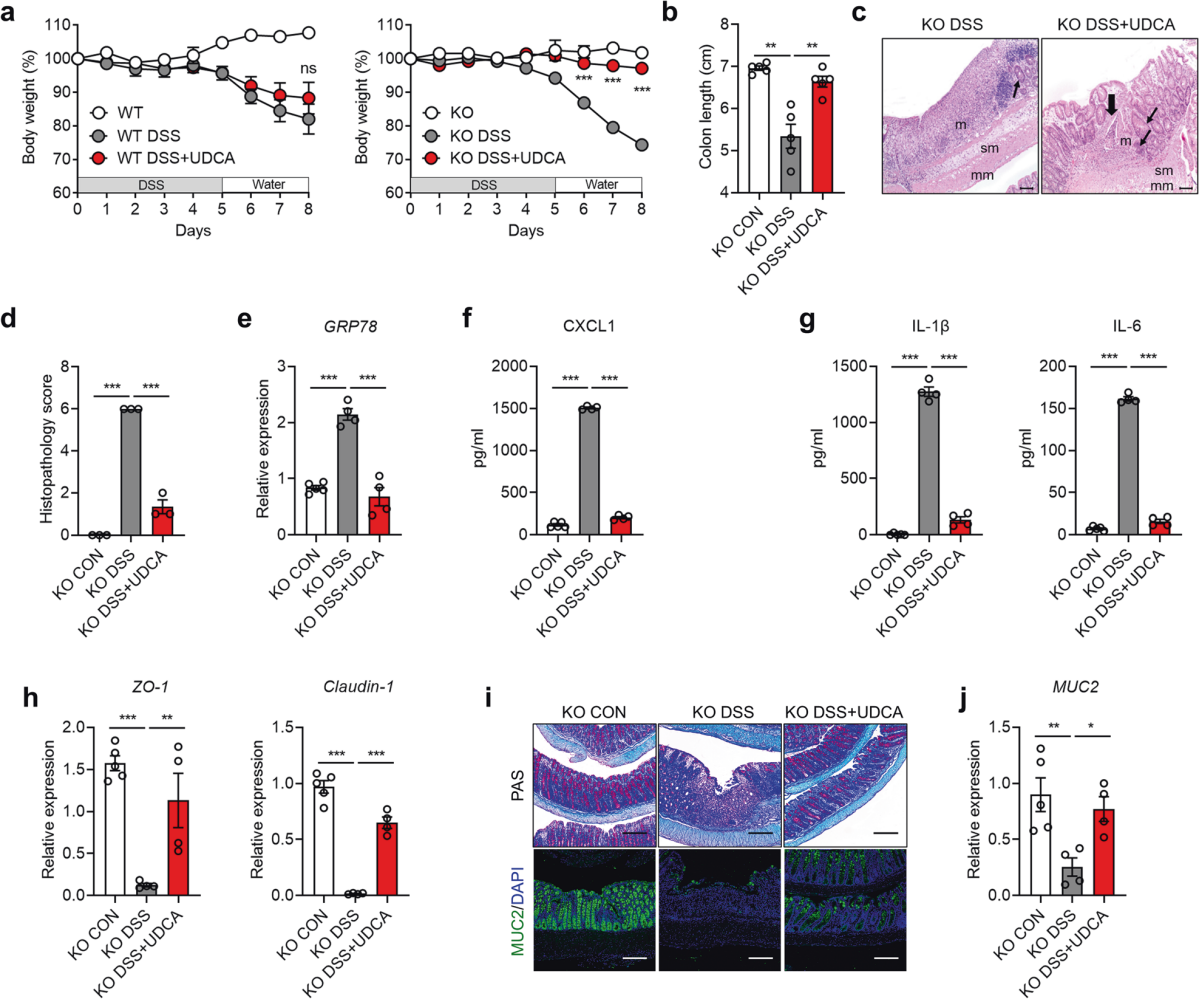
UDCA treatment ameliorates DSS-induced colitis in *ERdj5-*KO mice. WT and *ERdj5-*KO mice were administered 2% DSS in drinking water for 5 days and subsequently provided normal water. The mice were orally administered 500 mg/kg UDCA daily (*n* = 3–5 per group). **a** Bodyweight. **b** Colon length. **c** Representative H&E-stained colon tissue images (×100, scale bar 100 µm). Thin arrows indicate hyperplasia in affected areas. Thick arrows indicate multifocal inflammatory cell infiltration; m, mucosa; sm, submucosa; mm, muscular layer. **d** Histological scoring. **e** *GRP78* mRNA expression in colon tissues. **f** CXCL1, **g** IL-1 and IL-6 levels in colon homogenates. **h** *Zo-1* and *Cldn1* mRNA levels in colon tissues. **i** Representative PAS staining (×100) and MUC2 immunofluorescence (×200) images of the colon. Scale bar corresponds to 200 µm. **j** *MUC2* mRNA expression. The data are representative of three independent experiments, and the values are expressed as the mean ± SEM. *P < 0.05, **P < 0.01, ***P < 0.001; ns, not significant; one-way ANOVA was followed by Tukey’s test.

It has been previously reported that oral administration of UDCA protects against acute DSS-induced colitis^51^. Since ERdj5 deficiency increased ER stress and epithelial cell death, we postulated that treatment with chemical chaperones, including UDCA, could alleviate severe DSS-induced colitis in *ERdj5-*KO mice. Daily oral administration of UDCA to *ERdj5-*KO mice restored the lost body weight and shortened colon length induced by DSS administration (Fig. 6a, b). UDCA attenuated the severe infiltration of immune cells into the intestinal glands and tissue damage in DSS-treated *ERdj5-*KO mice (Fig. 6c, d). In addition, the transcription levels of *GRP78*, *XBP1s*, and *ATF4*, which were highly increased after DSS administration in *ERdj5-*KO mice, were reduced by UDCA treatment, relieving excessive ER stress (Fig. 6e, Supplementary Fig. 8d, e). Although DSS-administered *ERdj5-*KO mice treated with UDCA had restored IL-22 levels (Supplementary Fig. 8f), we believe that this UDCA-driven amelioration of DSS-induced colitis in *ERdj5-*KO mice was independent of the restoration of IL-22 levels (Supplementary Fig. 3d). In addition, the levels of CXCL1, IL-1β, and IL-6 were markedly reduced in DSS-administered *ERdj5-*KO mice following UDCA treatment (Fig. 6f, g). UDCA treatment also decreased neutrophil infiltration and restored tight junction proteins (Fig. 6h, Supplementary Fig. 3a). Finally, we observed that the reduced levels of MUC2 in DSS-administered *ERdj5-*KO mice were restored by UDCA treatment (Fig. 6i, j). Collectively, these results show that goblet cell dysfunction and subsequent severe inflammation in DSS-administered *ERdj5-*KO mice could be rescued by chemical chaperone-mediated ER stress reduction.

## Discussion

ERdj5 is highly expressed in the colon of patients with UC and in murine colitis. To investigate the role of ERdj5 in gut physiology and pathogenic colitis, we used *ERdj5-*KO mice. *ERdj5-*KO mice have a relatively normal gut physiology in the steady state. However, *ERdj5-*KO mice were more susceptible to DSS-induced colitis and enteric pathogens, such as *C. rodentium*, than WT mice. Although ablation of ERdj5 itself did not affect mucin production or goblet cell viability under homeotic conditions, there was a defect in mucin production under inflammatory conditions, presumably due to goblet cell apoptosis. In addition, ERdj5 deficiency weakened gut barrier integrity by reducing tight junction proteins and IL-22 production. Thus, reduced mucin production, increased goblet cell apoptosis, and damaged barrier integrity were observed in the colons of ERdj5-deficient mice following DSS administration or TLR stimulation. These findings suggest that ERdj5 is important for maintaining intestinal homeostasis by mucosal barriers under colitis conditions (Supplementary Fig. 10).

MUC2 has many cysteine residues linked by intra- and intermolecular disulfides for assembly in the ER and Golgi apparatus^52^. Disulfide bonds between the cysteine residues of mucin are important for proper folding and structural integrity^53,54^. MUC2 can serve as a substrate for ERdj5, which mediates the efficient cytosolic degradation of misfolded proteins via the ER-associated degradation pathway^55^. We determined that *ERdj5* was positively correlated with *MUC2*, *MUC1*, *MUC5AC*, and *MUC5B* (Supplementary Fig. 1a). In the colon of DSS-treated *ERdj5-*KO mice, the expression of *Galnt5* and *St3gal2*, which are involved in mucin-type O-glycan biosynthesis^56^, was downregulated, followed by reduced synthesis of mature mucin. Furthermore, the expression of *Hsph1*, *Hyou1*, and *Ero1l*, which are associated with protein folding in the ER^57^, was increased in the colons of *ERdj5-*KO mice, suggesting a critical need for chaperone activity during protein folding. In response to demand for MUC2 biosynthesis, ERdj5 deficiency disrupts the misfolded protein disposal system, leads to the accumulation of misfolded MUC2 and enhancing ER stress and the UPR. Based on the results from DSS-treated *ERdj5-*KO mice and *ERdj5-*KO MODE-K cells, ERdj5 deficiency increased ER stress, as evidenced by increased phosphorylation levels of PERK and eIF-2α, as well as GRP78, ATF4, and CHOP. CHOP is a transcription factor induced by ATF4 and cleaved ATF6 that induces several proapoptotic factors, including death receptor 5 (DR5) and growth arrest and DNA damage 34 (GADD34)^58^. Thus, the increased ER stress in *ERdj5-*KO mice with elevated CHOP expression might be responsible for the increased apoptosis of goblet cells induced by DSS administration and TLR stimulation. In addition, this finding suggests that ERdj5 regulates ER stress responses by correcting the misfolded MUC2 protein.

Goblet cells are easily exposed to ER stress under inflammatory and infectious conditions. Excessive ER stress can lead to the death of goblet cells, which is driven by ER stress-triggered reactive oxygen species (ROS)^59,60^. Inflammatory cytokines, including IL-1β, IL-6, and TNF-α, can stimulate mucin production^61^. In addition, some microbial products deliver innate signals that induce mucin production by IECs through NF-κB signaling^62^. DSS treatment also induces the hypersecretion of mucin^59^. When *ERdj5-*KO mice were exposed to environmental insults, such as DSS, TLR ligands, and infection, MUC2 production and goblet cells were markedly decreased in the colons of *ERdj5-*KO mice. Thus, ERdj5 is essential for maintaining goblet cell homeostasis under inflammatory conditions, although ERdj5 deficiency itself does not directly cause ER stress in vivo.

The importance of MUC2 production in gut barrier homeostasis has been revealed in several previous studies. Mice that were genetically deficient in MUC2 spontaneously developed severe intestinal inflammation, which frequently progressed to invasive adenocarcinoma and rectal tumors at a later age^63^. Similarly, Winnie and Eeyore mice, which contain missense mutations in MUC2, exhibit early dysbiosis, the loss of goblet cells, mucus barrier impairment, and spontaneous development of colitis^64^. The AGR2 and ER to nucleus signaling 2 (ERN2), which is also known as IRE1β^14,65^, are goblet cell-specific ER proteins involved in normal MUC2 biosynthesis. *Ire1β*^-/-^ mice have decreased production of intestinal MUC2 and increased susceptibility to DSS-induced colitis^66^. AGR2 variants have been identified as risk factors for IBD, including CD and UC^67^. AGR2 mediates the formation of disulfide bonds and proper folding of MUC2 through its thioredoxin-like domain^14^. Mice that are deficient in AGR2 are highly susceptible to DSS-induced colitis because of decreased levels of intestinal mucin, as well as abnormal Paneth cells in association with increased levels of ER stress^68^. In addition to genetic defects in MUC2 production, common food additive polysaccharides, such as maltodextrin, can trigger ER stress in goblet cells, mediate mucus depletion in association with alterations in MUC2 folding, and exacerbate chemically induced intestinal inflammation^69^. In infectious colitis and IBD, where MUC2 production is significantly increased, misfolded MUC2 accumulates, leading to ER stress-mediated goblet cell death^59^. These reports highlight the importance of proper MUC2 production for the maintenance of goblet cell health and overall mucosal homeostasis. Interestingly, although MUC2 protein levels were significantly reduced in *AGR2*^-/-^ mice and *MUC2*^-/-^ mice under steady-state conditions, *ERdj5-*KO mice exhibited normal levels of MUC2 in the colon^20,68^. In addition, there were no signs of defects in mucus-filled goblet cells of the colonic epithelium and colonoids from *ERdj5-*KO mice under steady-state conditions. These results suggest that ERdj5 is dispensable for MUC2 production in healthy gut environments. Thus, in the absence of ERdj5, other ER-resident PDI molecules, including AGR2 and PDIA3, may play compensatory roles to ensure proper ER function and proteostasis under steady-state conditions. Intriguingly, ERdj5 plays a crucial role in normal MUC2 biosynthesis. However, further investigations are necessary to elucidate the role of ERdj5 under inflammatory conditions.

ER stress and the UPR are important risk factors for IBD onset. As a consequence of the specific deletion of XBP-1 in murine IECs, aberrant ER stress leads to gut-specific inflammation^13,36^. Consistent with this finding, multiple single-nucleotide polymorphisms (SNPs) in XBP-1 have been observed in patients with IBD^13^. As common genetic factors in human IBD patients, SNPs recognize the UPR pathways, the PERK-ATF4-CHOP pathway, and GRP78, which is an important regulator of UPR initiation^13^. Activation of the NF-κB signaling pathway is a hallmark of intestinal inflammation in patients with IBD^70^. Gut microbiome-dependent TLR activation or an exacerbated ER stress pathway in IECs activates NF-κB signaling^36,71^. The depletion of XBP-1 in IECs results in IRE1-dependent NF-κB activation^36^, which might be triggered by TLR-dependent signaling in response to microbial substances^71^. Therefore, we postulated that ER stress in IECs was closely associated with intestinal inflammation. Accordingly, several previous reports have suggested that ER stress can reinforce the NF-κB signaling pathway. Chemically induced ER stress with tunicamycin or thapsigargin induces NF-κB activation in an IRE1-dependent manner^72^, and TRAF2 plays a critical role in ER stress-induced IκBα degradation and NF-kB activation^73^. In addition, the PERK/eIF2α pathway, another UPR axis, is associated with NF-κB activation under ER stress conditions^74^. Phosphorylation of eIF2α activates the NF-κB pathway by suppressing the translation of IκBα without inducing IκB degradation^75^. Finally, the ATF6 branch of the UPR is associated with NF-κB activation in response to ER stress. Inhibition of ATF6 suppresses NF-κB activation by Shiga-toxigenic *E. coli* toxin^76^. In the present study, ERdj5 deficiency promoted ER stress and reinforced TLR2-induced NF-κB activation, followed by enhanced chemokine production. Collectively, these results suggest that the attenuation of NF-κB could be a successful therapeutic approach for ER stress-associated inflammatory diseases, including IBD.

Conversely, inflammation can also induce an ER stress response. ER stress is known to be associated with inflammatory conditions, such as atherosclerosis, cystic fibrosis, and IBD^15,77,78^. As an example of inflammation-induced ER stress, proinflammatory cytokines, including IL-6, IL-1β, and TNF-α, can trigger the UPR^74^. Furthermore, inflammation-induced ROS and nitric oxide (NO) production, as well as inhibition of sarco/endoplasmic reticulum Ca^2+^ ATPase (SERCA) and ER chaperones, are also associated with the UPR^79^. Thus, inflammation itself can induce the ER stress response, and in turn, ER stress can enhance the inflammatory response to TLR stimulation. To develop preventive and therapeutic drugs against IBD, several strategies that reduce ER stress have been effective in preclinical studies. Recently, chemical chaperones, including UDCA and 4-phenylbutyric acid, were shown to suppress gut inflammation in a mouse model^80^. We confirmed that UDCA treatment significantly improved DSS-induced colitis in *ERdj5-*KO mice, suggesting that UDCA is a potential therapeutic candidate for the treatment of ER stress-related colitis.

Defective degradation of misfolded MUC2 in the context of ERdj5 deficiency leads to defective mucus barrier integrity and inflammation, which increases ER stress. The increased ER stress caused by ERdj5 deficiency affects goblet cells more severely, leading to their selective depletion by apoptosis. Our findings highlight the crucial role of ERdj5 in preserving goblet cell survival and function, as well as the importance of the mucus barrier in maintaining gut homeostasis. UDCA treatment of DSS-administered *ERdj5-*KO mice ameliorated colitis by restoring mucin production and gut barrier molecules, suggesting the potential of UDCA in the treatment of patients with IBD. These results suggest that the amelioration of excessive ER stress could be a prospective strategy for the development of preventive and therapeutic agents for the treatment of patients with IBD.

## Supplementary information

Supplementary_Figure

## References

[1] McCracken VJ, Lorenz RG (2001). The gastrointestinal ecosystem: a precarious alliance among epithelium, immunity and microbiota. Cell. Microbiol..

[2] Liévin-Le Moal V, Servin AL (2006). The front line of enteric host defense against unwelcome intrusion of harmful microorganisms: mucins, antimicrobial peptides, and microbiota. Clin. Microbiol. Rev..

[3] Johansson ME, Hansson GC (2016). Immunological aspects of intestinal mucus and mucins. Nat. Rev. Immunol..

[4] van Putten JPM, Strijbis K (2017). Transmembrane Mucins: Signaling Receptors at the Intersection of Inflammation and Cancer. J. Innate Immun..

[5] Pelaseyed T (2014). The mucus and mucins of the goblet cells and enterocytes provide the first defense line of the gastrointestinal tract and interact with the immune system. Immunol. Rev..

[6] Fang J (2021). Slimy partners: the mucus barrier and gut microbiome in ulcerative colitis. Exp. Mol. Med..

[7] Singh V (2022). Chronic Inflammation in Ulcerative Colitis Causes Long-Term Changes in Goblet Cell Function. Cell. Mol. Gastroenterol. Hepatol..

[8] Depaepe T (2021). At the Crossroads of Survival and Death: The Reactive Oxygen Species-Ethylene-Sugar Triad and the Unfolded Protein Response. Trends Plant Sci..

[9] Kaser A, Blumberg RS (2010). Survive an innate immune response through XBP1. Cell Res..

[10] Gardner BM, Pincus D, Gotthardt K, Gallagher CM, Walter P (2013). Endoplasmic reticulum stress sensing in the unfolded protein response. Cold Spring Harb. Perspect. Biol..

[11] Lee AH, Chu GC, Iwakoshi NN, Glimcher LH (2005). XBP-1 is required for biogenesis of cellular secretory machinery of exocrine glands. Embo J..

[12] Kaser A, Zeissig S, Blumberg RS (2010). Inflammatory bowel disease. Annu. Rev. Immunol..

[13] Kaser A (2008). XBP1 links ER stress to intestinal inflammation and confers genetic risk for human inflammatory bowel disease. Cell.

[14] Park SW (2009). The protein disulfide isomerase AGR2 is essential for production of intestinal mucus. Proc. Natl Acad. Sci. U. S. A..

[15] Kaser A, Martínez-Naves E, Blumberg RS (2010). Endoplasmic reticulum stress: implications for inflammatory bowel disease pathogenesis. Curr. Opin. Gastroenterol..

[16] Meng Y (2015). The protein disulfide isomerase 1 of Phytophthora parasitica (PpPDI1) is associated with the haustoria-like structures and contributes to plant infection. Front. Plant Sci..

[17] Haeri M, Knox BE (2012). Endoplasmic Reticulum Stress and Unfolded Protein Response Pathways: Potential for Treating Age-related Retinal Degeneration. J. Ophthalmic Vis. Res..

[18] Sato Y (2013). Synergistic cooperation of PDI family members in peroxiredoxin 4-driven oxidative protein folding. Sci. Rep..

[19] Linden SK, Sutton P, Karlsson NG, Korolik V, McGuckin MA (2008). Mucins in the mucosal barrier to infection. Mucosal Immunol..

[20] Van der Sluis M (2006). Muc2-deficient mice spontaneously develop colitis, indicating that MUC2 is critical for colonic protection. Gastroenterology.

[21] Al-Shaibi AA (2021). Human AGR2 Deficiency Causes Mucus Barrier Dysfunction and Infantile Inflammatory Bowel Disease. Cell. Mol. Gastroenterol. Hepatol..

[22] Ushioda R (2016). Redox-assisted regulation of Ca2+ homeostasis in the endoplasmic reticulum by disulfide reductase ERdj5. Proc. Natl Acad. Sci. U. S. A..

[23] Oka OB, Pringle MA, Schopp IM, Braakman I, Bulleid NJ (2013). ERdj5 is the ER reductase that catalyzes the removal of non-native disulfides and correct folding of the LDL receptor. Mol. Cell.

[24] Muñoz-Lobato F (2014). Protective role of DNJ-27/ERdj5 in Caenorhabditis elegans models of human neurodegenerative diseases. Antioxid. Redox Signal..

[25] Apostolou E, Moustardas P, Iwawaki T, Tzioufas AG, Spyrou G (2019). Ablation of the Chaperone Protein ERdj5 Results in a Sjögren’s Syndrome-Like Phenotype in Mice, Consistent With an Upregulated Unfolded Protein Response in Human Patients. Front. Immunol..

[26] Hosoda A, Tokuda M, Akai R, Kohno K, Iwawaki T (2009). Positive contribution of ERdj5/JPDI to endoplasmic reticulum protein quality control in the salivary gland. Biochem. J..

[27] Huang da W, Sherman BT, Lempicki RA (2009). Bioinformatics enrichment tools: paths toward the comprehensive functional analysis of large gene lists. Nucleic Acids Res..

[28] Huang da W, Sherman BT, Lempicki RA (2009). Systematic and integrative analysis of large gene lists using DAVID bioinformatics resources. Nat. Protoc..

[29] Wang Y (2020). Single-cell transcriptome analysis reveals differential nutrient absorption functions in human intestine. J. Exp. Med..

[30] Kim YI (2018). CX(3)CR1(+) Macrophages and CD8(+) T Cells Control Intestinal IgA Production. J. Immunol..

[31] Graves CL (2014). A method for high purity intestinal epithelial cell culture from adult human and murine tissues for the investigation of innate immune function. J. Immunol. Methods.

[32] Erben U (2014). A guide to histomorphological evaluation of intestinal inflammation in mouse models. Int. J. Clin. Exp. Pathol..

[33] Allaire JM (2021). Interleukin-37 regulates innate immune signaling in human and mouse colonic organoids. Sci. Rep..

[34] Grebenyuk S, Ranga A (2019). Engineering Organoid Vascularization. Front. Bioeng. Biotechnol..

[35] Taylor GA (2020). Irgm1-deficiency leads to myeloid dysfunction in colon lamina propria and susceptibility to the intestinal pathogen Citrobacter rodentium. PLoS Pathog..

[36] Adolph TE (2013). Paneth cells as a site of origin for intestinal inflammation. Nature.

[37] Sanjana NE, Shalem O, Zhang F (2014). Improved vectors and genome-wide libraries for CRISPR screening. Nat. Methods.

[38] Cunnea PM (2003). ERdj5, an endoplasmic reticulum (ER)-resident protein containing DnaJ and thioredoxin domains, is expressed in secretory cells or following ER stress. J. Biol. Chem..

[39] Sroussi HY, Lu Y, Zhang QL, Villines D, Marucha PT (2010). S100A8 and S100A9 inhibit neutrophil oxidative metabolism in-vitro: involvement of adenosine metabolites. Free Radic. Res..

[40] Zhang G (2018). Elevated Expression of Serum Amyloid A 3 Protects Colon Epithelium Against Acute Injury Through TLR2-Dependent Induction of Neutrophil IL-22 Expression in a Mouse Model of Colitis. Front. Immunol..

[41] Zhang X, Xu W (2017). Neutrophils diminish T-cell immunity to foster gastric cancer progression: the role of GM-CSF/PD-L1/PD-1 signalling pathway. Gut.

[42] Yadav S (2018). Nitric oxide synthase 2 enhances the survival of mice during Salmonella Typhimurium infection-induced sepsis by increasing reactive oxygen species, inflammatory cytokines and recruitment of neutrophils to the peritoneal cavity. Free Radic. Biol. Med..

[43] Lee B, Moon KM, Kim CY (2018). Tight Junction in the Intestinal Epithelium: Its Association with Diseases and Regulation by Phytochemicals. J. Immunol. Res..

[44] Groschwitz KR, Hogan SP (2009). Intestinal barrier function: molecular regulation and disease pathogenesis. J. Allergy Clin. Immunol..

[45] Wei HX, Wang B, Li B (2020). IL-10 and IL-22 in Mucosal Immunity: Driving Protection and Pathology. Front. Immunol..

[46] Kim S (2022). Amelioration of DSS-Induced Acute Colitis in Mice by Recombinant Monomeric Human Interleukin-22. Immune Netw..

[47] Sham HP (2018). Immune Stimulation Using a Gut Microbe-Based Immunotherapy Reduces Disease Pathology and Improves Barrier Function in Ulcerative Colitis. Front. Immunol..

[48] Bergstrom KS (2008). Modulation of intestinal goblet cell function during infection by an attaching and effacing bacterial pathogen. Infect. Immun..

[49] Thomas CG, Spyrou G (2009). ERdj5 sensitizes neuroblastoma cells to endoplasmic reticulum stress-induced apoptosis. J. Biol. Chem..

[50] Kim S, Joe Y, Surh YJ, Chung HT (2018). Differential Regulation of Toll-Like Receptor-Mediated Cytokine Production by Unfolded Protein Response. Oxid. Med. Cell. Longev..

[51] Van den Bossche L (2017). Ursodeoxycholic Acid and Its Taurine- or Glycine-Conjugated Species Reduce Colitogenic Dysbiosis and Equally Suppress Experimental Colitis in Mice. Appl. Environ. Microbiol..

[52] Javitt G (2020). Assembly Mechanism of Mucin and von Willebrand Factor Polymers. Cell.

[53] Mueller P (2018). High level in vivo mucin-type glycosylation in Escherichia coli. Microb. Cell Fact..

[54] Cornick S, Tawiah A, Chadee K (2015). Roles and regulation of the mucus barrier in the gut. Tissue Barriers.

[55] Ushioda R (2008). ERdj5 is required as a disulfide reductase for degradation of misfolded proteins in the ER. Science.

[56] Xi D (2021). The glycosyltransferase ST3GAL2 is regulated by miR-615-3p in the intestinal tract of Campylobacter jejuni infected mice. Gut Pathog..

[57] Piróg KA (2019). XBP1 signalling is essential for alleviating mutant protein aggregation in ER-stress related skeletal disease. PLoS Genet..

[58] Hu H, Tian M, Ding C, Yu S (2018). The C/EBP Homologous Protein (CHOP) Transcription Factor Functions in Endoplasmic Reticulum Stress-Induced Apoptosis and Microbial Infection. Front. Immunol..

[59] Tawiah A (2018). High MUC2 Mucin Expression and Misfolding Induce Cellular Stress, Reactive Oxygen Production, and Apoptosis in Goblet Cells. Am. J. Pathol..

[60] Tiwari S, Begum S, Moreau F, Gorman H, Chadee K (2021). Autophagy is required during high MUC2 mucin biosynthesis in colonic goblet cells to contend metabolic stress. Am. J. Physiol. Gastrointest. Liver Physiol..

[61] Enss ML (2000). Proinflammatory cytokines trigger MUC gene expression and mucin release in the intestinal cancer cell line LS180. Inflamm. Res..

[62] Lemjabbar H, Basbaum C (2002). Platelet-activating factor receptor and ADAM10 mediate responses to Staphylococcus aureus in epithelial cells. Nat. Med..

[63] Velcich A (2002). Colorectal cancer in mice genetically deficient in the mucin Muc2. Science.

[64] Heazlewood CK (2008). Aberrant mucin assembly in mice causes endoplasmic reticulum stress and spontaneous inflammation resembling ulcerative colitis. PLoS Med..

[65] Tsuru A (2013). Negative feedback by IRE1β optimizes mucin production in goblet cells. Proc. Natl Acad. Sci. U. S. A..

[66] Bertolotti A (2001). Increased sensitivity to dextran sodium sulfate colitis in IRE1beta-deficient mice. J. Clin. Invest..

[67] Zheng W (2006). Evaluation of AGR2 and AGR3 as candidate genes for inflammatory bowel disease. Genes Immun..

[68] Zhao F (2010). Disruption of Paneth and goblet cell homeostasis and increased endoplasmic reticulum stress in Agr2-/- mice. Dev. Biol..

[69] Laudisi F (2019). The Food Additive Maltodextrin Promotes Endoplasmic Reticulum Stress-Driven Mucus Depletion and Exacerbates Intestinal Inflammation. Cell. Mol. Gastroenterol. Hepatol..

[70] Liu T, Zhang L, Joo D, Sun SC (2017). NF-κB signaling in inflammation. Signal Transduct. Target. Ther..

[71] Martinon F, Chen X, Lee AH, Glimcher LH (2010). TLR activation of the transcription factor XBP1 regulates innate immune responses in macrophages. Nat. Immunol..

[72] Hu P, Han Z, Couvillon AD, Kaufman RJ, Exton JH (2006). Autocrine tumor necrosis factor alpha links endoplasmic reticulum stress to the membrane death receptor pathway through IRE1alpha-mediated NF-kappaB activation and down-regulation of TRAF2 expression. Mol. Cell. Biol..

[73] Kaneko M, Niinuma Y, Nomura Y (2003). Activation signal of nuclear factor-kappa B in response to endoplasmic reticulum stress is transduced via IRE1 and tumor necrosis factor receptor-associated factor 2. Biol. Pharm. Bull..

[74] Chipurupalli S, Samavedam U, Robinson N (2021). Crosstalk Between ER Stress, Autophagy and Inflammation. Front. Med. (Lausanne).

[75] Deng J (2004). Translational repression mediates activation of nuclear factor kappa B by phosphorylated translation initiation factor 2. Mol. Cell. Biol..

[76] Yamazaki H (2009). Activation of the Akt-NF-kappaB pathway by subtilase cytotoxin through the ATF6 branch of the unfolded protein response. J. Immunol..

[77] Hotamisligil GS (2010). Endoplasmic reticulum stress and atherosclerosis. Nat. Med..

[78] Ribeiro CM, Boucher RC (2010). Role of endoplasmic reticulum stress in cystic fibrosis-related airway inflammatory responses. Proc. Am. Thorac. Soc..

[79] Ly LD (2017). Oxidative stress and calcium dysregulation by palmitate in type 2 diabetes. Exp. Mol. Med..

[80] Cao SS (2013). The unfolded protein response and chemical chaperones reduce protein misfolding and colitis in mice. Gastroenterology.

