# Supplementary material for: ERdj5 protects goblet cells from endoplasmic reticulum stress-mediated apoptosis under inflammatory conditions
Source: Exp Mol Med. 2023 Feb 9;55(2):401–12. doi: 10.1038/s12276-023-00945-x (PMC9981579; doi:10.1038/s12276-023-00945-x)
Supplement: Supplementary file 1 — Supplementary_Figure [file 12276_2023_945_MOESM1_ESM.pdf]

Supplementary information

ERdj5 protects goblet cells from endoplasmic reticulum stress-mediated apoptosis under inflammatory conditions

Hyunjin Jeong[1,2*], Eun-Hye Hong[1*], Jae-Hee Ahn[1,2], Jaewon Cho[1,2], Jae-Hyeon Jeong[1,2], Chae-Won Kim[1,2], Byung-Il Yoon[3], Ja Hyun Koo[4], Yun-Yong Park[5], Yoon Mee Yang[1,2], Takao Iwawaki[6], Bruce A. Vallance[7], Sun-Young Chang[8] and Hyun-Jeong Ko[1,9]

This file includes **Supplementary Figures 1–10**.

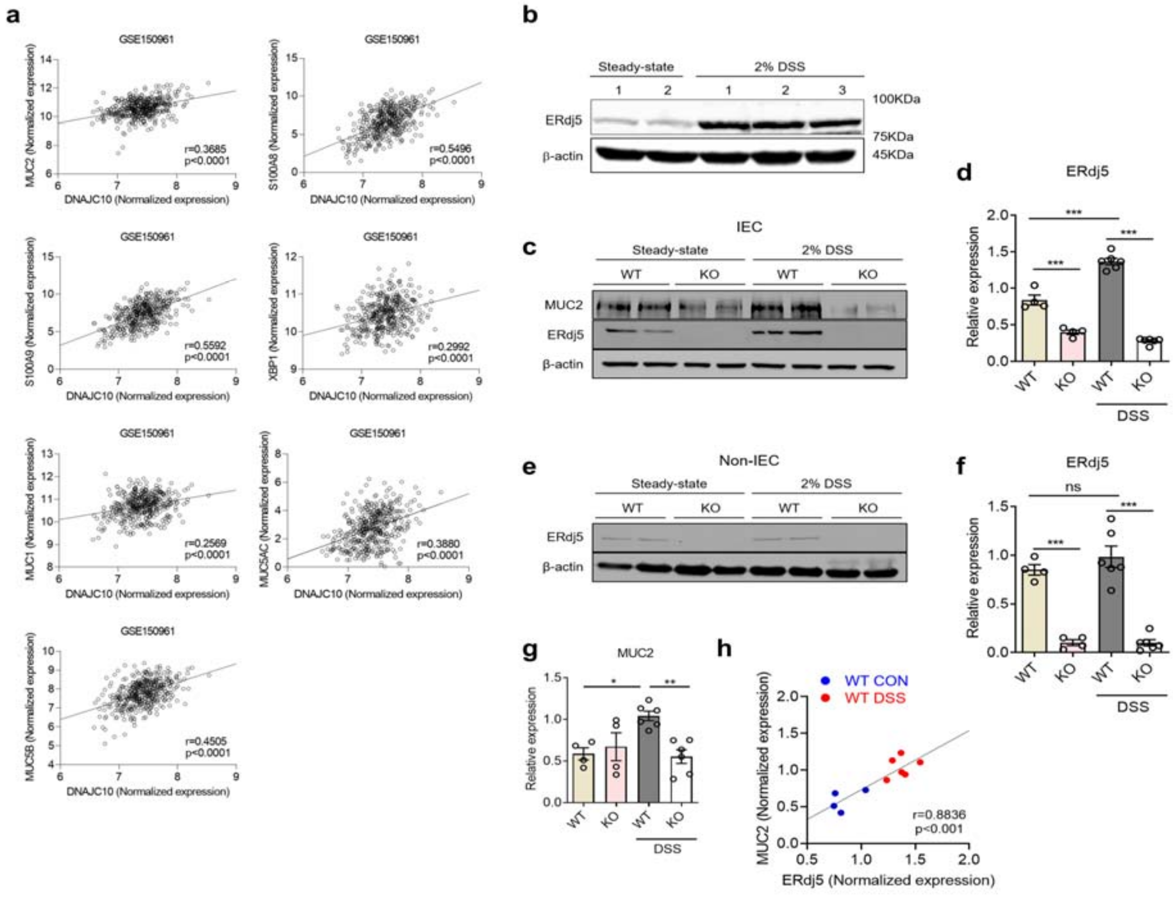

Supplementary Fig. 1. Augmented ERdj5 expression in inflamed colons of humans and mice.

(a) Correlations among the mRNA expression profiles of *ERdj5, Muc2*, *S100A8*, *S100A9*, *Xbp-1, Muc1*, *Muc5ac*, and *Muc5b*. (b) WT mice administered water containing 2% DSS for 5 days followed by normal water for 3 days. On day 8, the protein expression level of ERdj5 was analyzed in the colon. (c) ERdj5 and MUC-2 protein expression and (d) *ERdj5* mRNA expression in IEC isolated from colon tissues (n=4-6 per group). (e) ERdj5 protein expression and (f) *ERdj5* mRNA expression in non-IEC obtained from the colon tissues (n=4-6 per group). (g) *MUC2* mRNA expression in IEC from colon tissues (n=4-6 per group). (h) Correlation analysis of *MUC2* and *ERdj5* mRNA expression in IEC from WT CON and WT DSS (n=4-6 per group). $^*P < 0.05$, $^{**}P < 0.01$, $^{***}P < 0.001$; ns, not significant; one-way ANOVA followed by Tukey's test

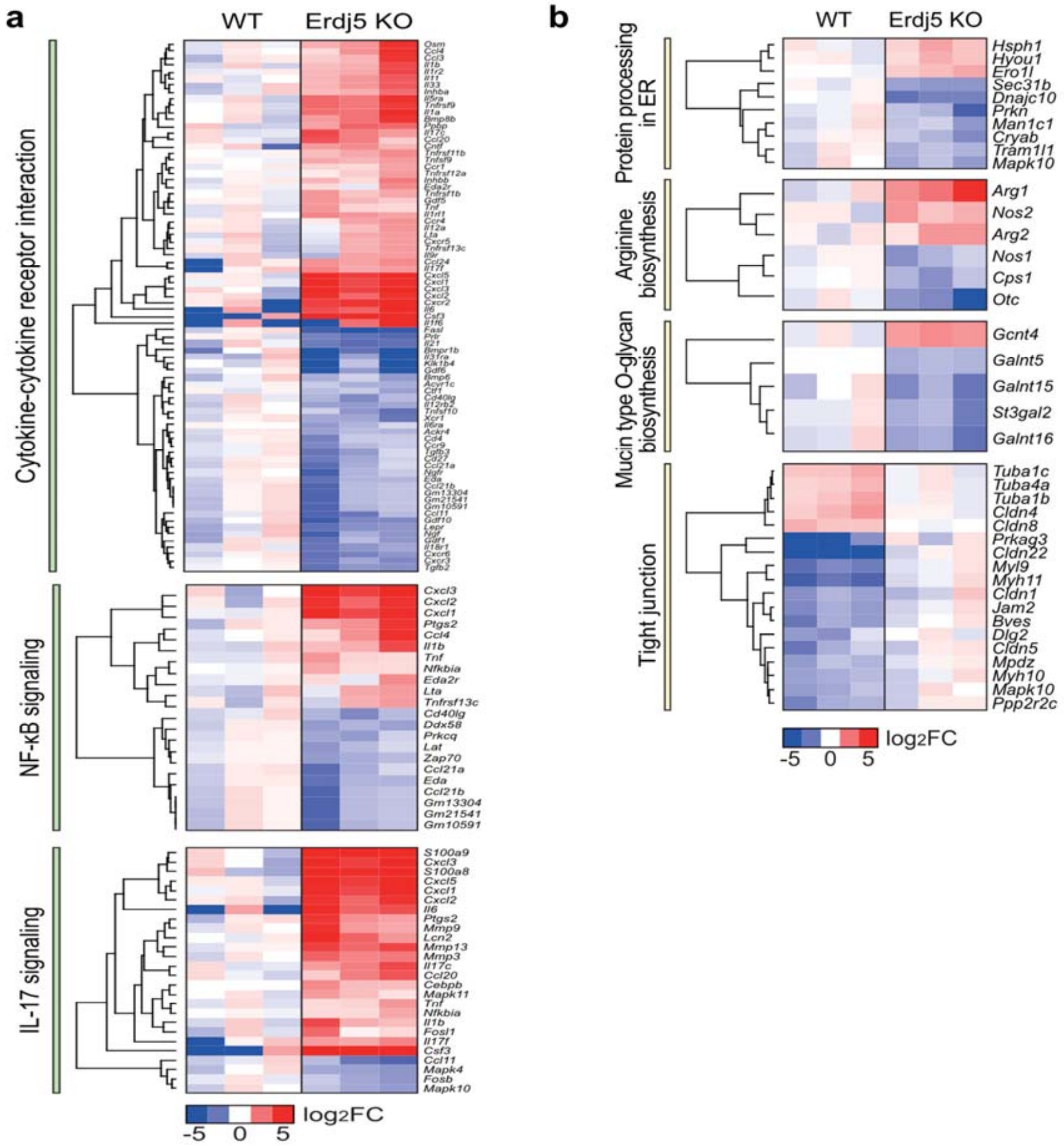

Supplementary Fig. 2. Several gene expression profiles associated with inflammation and gut barrier function are altered in the colons of ERdj5-KO mice.

(a) Heatmap of the DEGs associated with cytokine-cytokine receptor interaction, NF-κB signaling, and IL-17 signaling. (b) Heatmap of DEGs associated with protein processing in ER, arginine biosynthesis, mucin-type O-glycan biosynthesis, and tight junction goblet cell function-related protein folding and mucin biosynthesis. The $\log_2$ ratios of *ERdj5* KO DSS/WT DSS presented in the heatmap (blue, under-expression; red, over-expression). Values over 5 or under -5 were rounded.

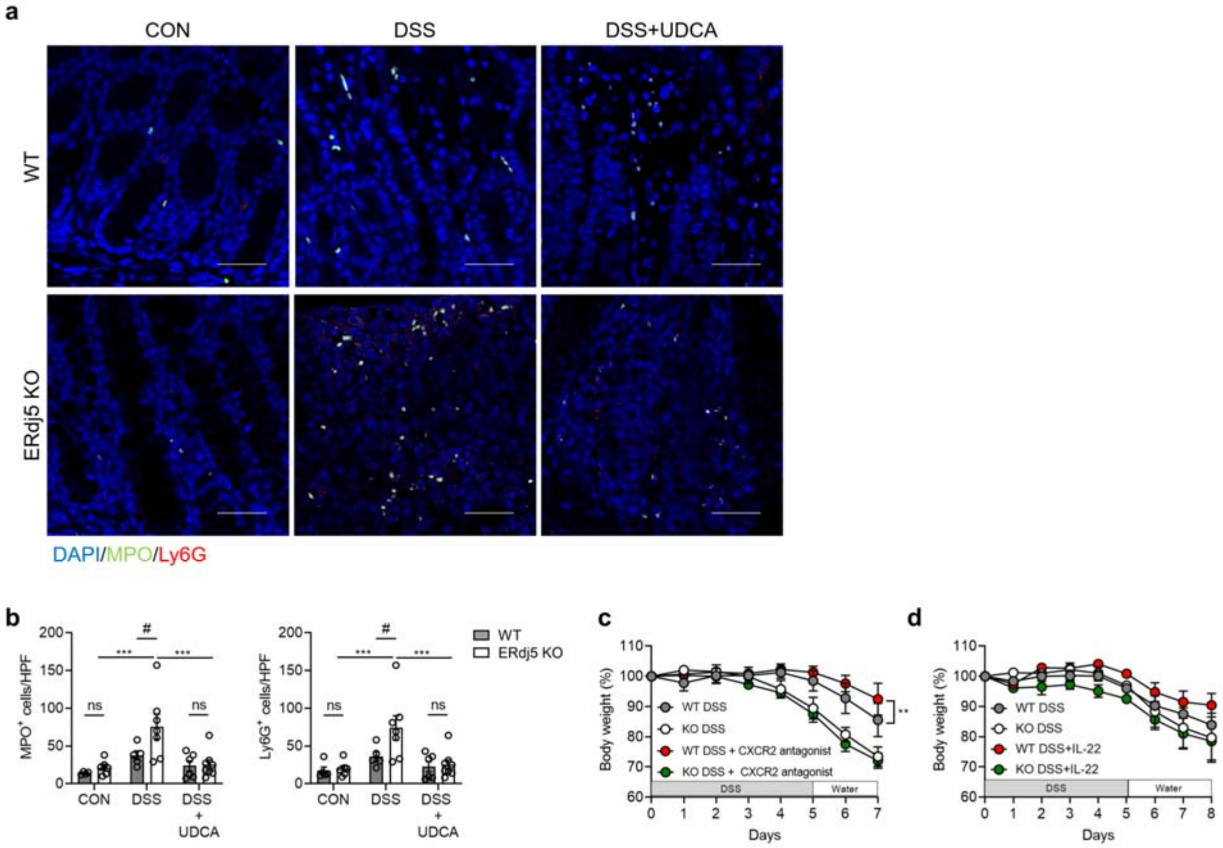

Supplementary Fig. 3. DSS-mediated colitis induces excessive neutrophil infiltration in the colons of *ERdj5* KO mice, which is improved by UDCA administration but not by CXCR2 antagonist treatment.

WT and *ERdj5* KO mice administered 2% DSS in drinking water for 5 days and then with normal water. Mice were orally administered with 500 mg/kg UDCA (n=5 per group), i.p. injected with 1 mg/kg CXCR2 antagonist daily (n=4 per group) or intraperitoneally. injected with 50 µg/kg recombinant mouse IL-22 every other day (n=3 per group). (a) Representative immunofluorescence images of MPO (green), Ly6G (red), and DAPI (blue) (×200, scale bar 100 µm) in mice colons. (b) MPO^+ and $Ly6G^+$ cells, indicating neutrophils, were counted (n=4-8 per group). (c, d) Body weight. *P < 0.05, **P < 0.01, ***P < 0.001, # vs control; ns, not significant; one-way ANOVA followed by Tukey's test. HPF, high-power field; MPO, myeloperoxidase.

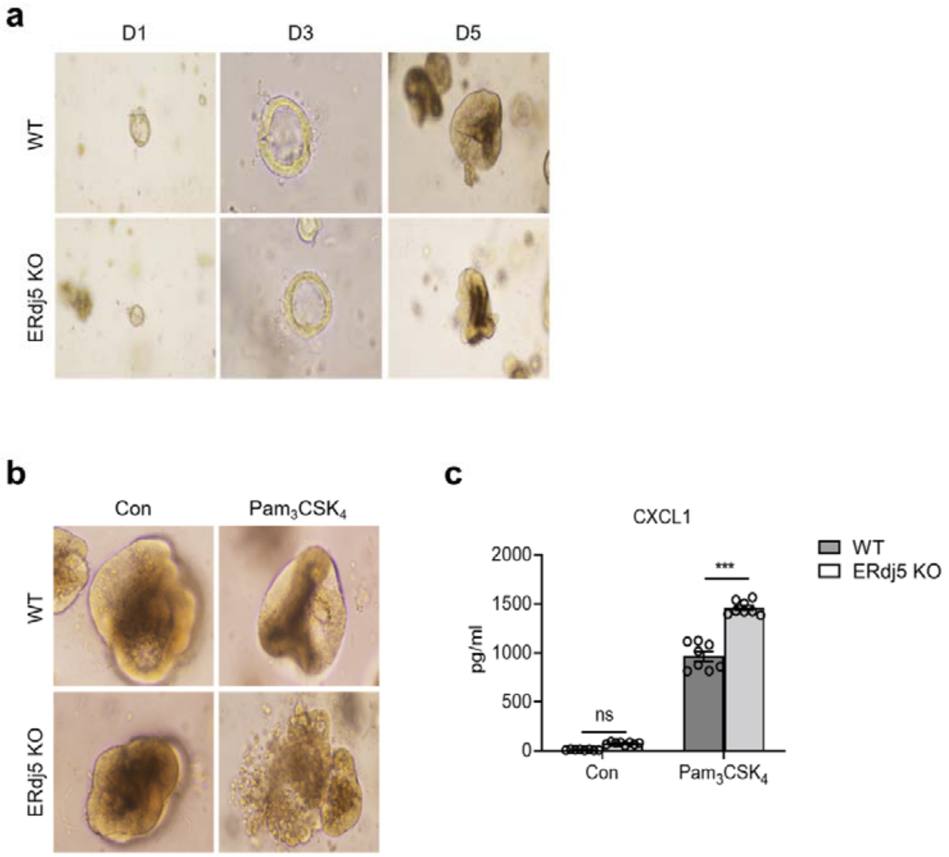

Supplementary Fig. 4. ERdj5-deficient goblet cells are more susceptible to apoptosis induced by TLR2 stimulation.

Intestinal crypts isolated from the colons of *ERdj5* WT and *ERdj5* KO mice and cultured in Matrigel. (a) Representative images showing the growth pattern of colon organoids on days 1, 3, and 5. (b) Representative bright-field images (×400) and (c) CXCL1 production in colon organoids at day 7, which were treated with Pam_3CSK_4 (1 μg/ml) for 48 h (n=8 per group). ***$P < 0.001$; ns, not significant; one-way ANOVA followed by Tukey's test.

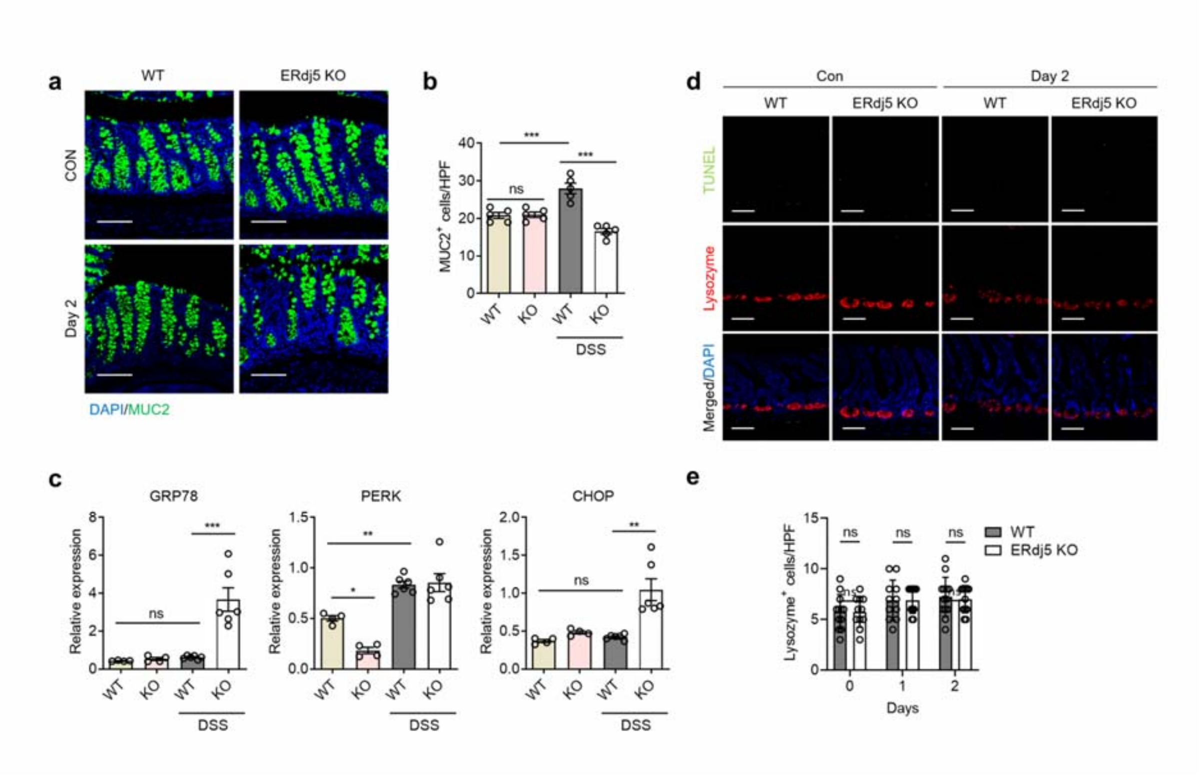

Supplementary Fig. 5. ERdj5 deficiency mediates early onset of colonic goblet cells dysfunction but does not alter Paneth cells in the small intestine on day 2 after DSS treatment.

(a) Representative immunofluorescence images of colon tissues from WT and *ERdj5* KO mice on day 2 after DSS treatment. MUC2 (green) and DAPI (blue) (×200, scale bar 100 μm). (b) $MUC2^+$ cell count per high-power field (HPF) from the colon tissue images (n=5 per group). (c) mRNA levels of *GRP78, PERK,* and *CHOP* (n=4-6 per group). (d) On day 2 following 2% DSS treatment, $lysozyme^+$ Paneth cells in the small intestine of *ERdj5* WT and KO mice were visualized by immunofluorescence staining (×400, scale bar 100 μm). (e) Number of $lysozyme^+$ Paneth cells per crypt (n=11-13 per group). *$P < 0.05$, **$P < 0.01$, ***$P < 0.001$; ns, not significant; one-way ANOVA followed by Tukey's test

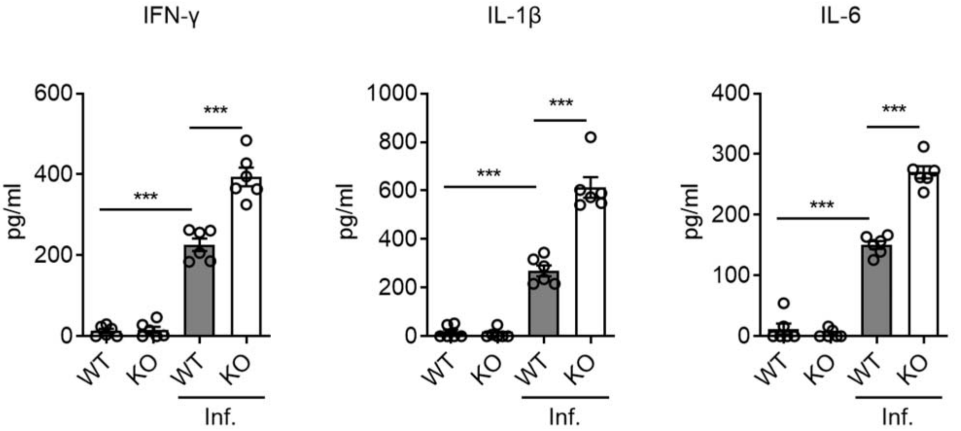

Supplementary Fig. 6. ERdj5 deficiency aggravates inflammation after *C. rodentium* infection.

Levels of IFN-γ, IL-1β and IL-6 in colon homogenates of WT or *ERdj5* KO mice 14 days after *C. rodentium* infection (n=6 per group). ***P < 0.001; one-way ANOVA followed by Tukey’s test.

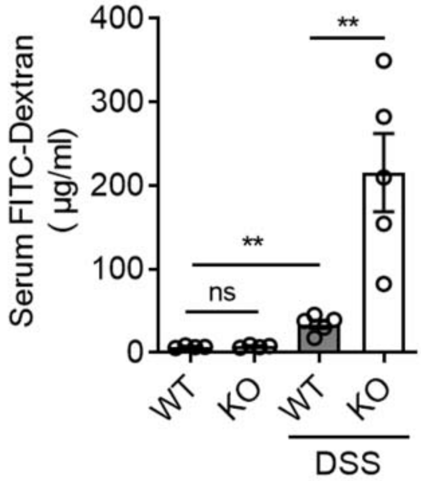

Supplementary Fig. 7. ERdj5 deficiency exacerbates intestinal permeability

Barrier function assessed by FITC-dextran permeability in WT or *ERdj5* KO mice on day 8 following DSS treatment. (n=4-5 per group). **P < 0.01; ns, not significant; one-way ANOVA followed by Tukey’s test.

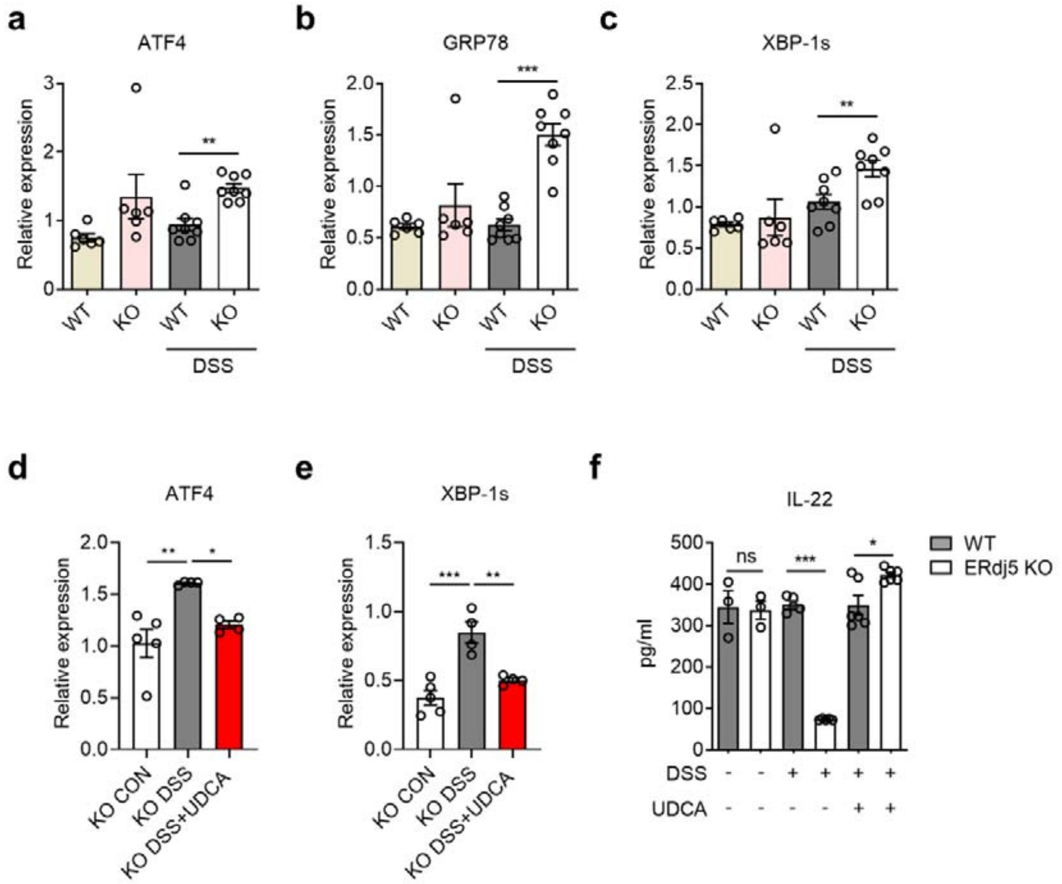

Supplementary Fig. 8. ERdj5 deficiency increases mRNA expression of several genes associated with ER stress, which was decreased by UDCA treatment.

mRNA transcription levels of (a) *ATF4*, (b) *GRP78/BiP*, and (c) *XBP1s* in the colons of WT or *ERdj5* KO mice treated with DSS (n=6-8 per group). Administration of UDCA decreased the levels of (d) *ATF4* and (e*) XBP1s* mRNA expression in the colons of *ERdj5* KO mice (n=4-5 per group). (f) IL-22 expression in colon tissue homogenates (n=3-6 per group). *P < 0.05, **P < 0.01, ***P < 0.001; ns, not significant; one-way ANOVA followed by the Tukey's test.

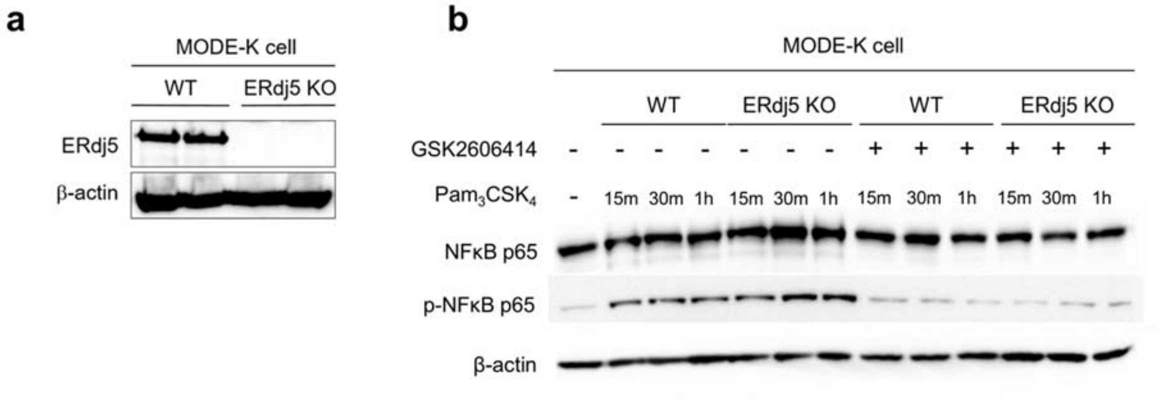

Supplementary Fig. 9. ERdj5 knock out in MODE-K cells using the CRISPR/Cas9 system. (a) The expression of ERdj5 was blunted in MODE-K cells using CRISPR/Cas9, which was confirmed by western blotting. (b) MODE-K cells were treated with 5 μM GSK 2606414, a PERK inhibitor, for 2 hr before Pam3CSK4 treatment.

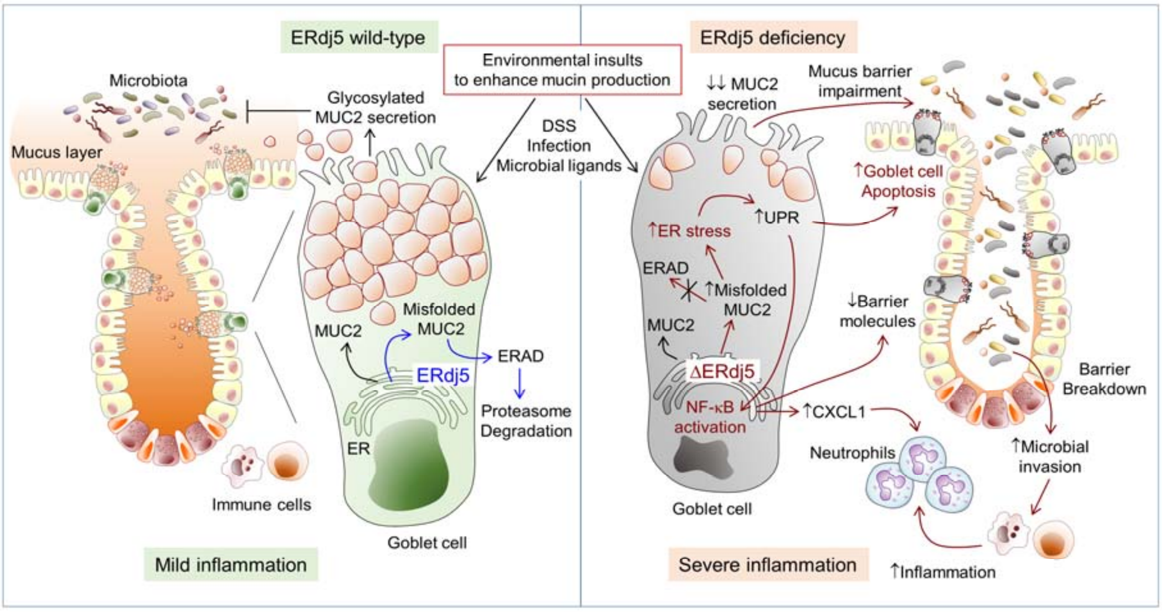

Supplementary Fig. 10. Summary figure.

Schematic summary of the role of ERdj5 in gut barrier function under environmental stimuli. *ERdj5* KO mice have normal gut physiology at a steady-state, but inflammatory stimuli, such as DSS or pathogens, induce more severe colitis. ERdj5 deficiency induces ER stress due to accumulation of misfolded MUC2, which in turn leads to early apoptosis of goblet cells. ERdj5 deficiency weakens gut barrier integrity in association with NF-κB pathway activation. Thus, reduced mucin production, increased apoptosis of goblet cells, and damaged barrier integrity in the colons of *ERdj5*-deficient mice following DSS administration or TLR stimulation demonstrate that ERdj5 plays a crucial role in maintaining intestinal homeostasis.
